# Shifting Antarctic Circumpolar Current south of Africa over the past 1.9 million years

**DOI:** 10.1126/sciadv.adp1692

**Published:** 2025-01-01

**Authors:** Aidan Starr, Ian R. Hall, Stephen Barker, Alexandra Nederbragt, Lindsey Owen, Sidney R. Hemming

**Affiliations:** ^1^ School of Earth and Environmental Sciences, Cardiff University, Cardiff CF10 3AT, UK.; ^2^ Lamont-Doherty Earth Observatory, Columbia University, Palisades, NY 10964, USA.

## Abstract

The Antarctic Circumpolar Current (ACC) dominates the transfer of heat, salt, and tracers around the Southern Ocean (SO), driving the upwelling of carbon-rich deep waters around Antarctica. Paleoclimate reconstructions reveal marked variability in SO circulation; however, few records exist coupling quantitative reconstructions of ACC flow with tracers of SO upwelling spanning multiple Pleistocene glacial cycles. Here, we reconstruct near-bottom flow speed variability in the SO south of Africa, revealing systematic glacial-interglacial variations in the strength and/or proximity of ACC jets. These are superimposed by warmer-than-present “super-interglacials,” whereby extreme slowdown in the midlatitude ACC (41°S) is opposed by faster flow at higher latitudes (>54°S), implying poleward strengthening of the ACC. Coupled with reconstructions of the subsurface-deep stable carbon isotope gradient, we show that the reorganization of ACC coincides with the upwelling of isotopically light deep waters around Antarctica, likely contributing to the interglacial rise in atmospheric carbon dioxide (CO_2_) levels.

## Teaser

Shifts in the world’s largest ocean current are closely linked to Southern Ocean upwelling during warm intervals in Earth’s past.

## INTRODUCTION

**Fig. 1.**
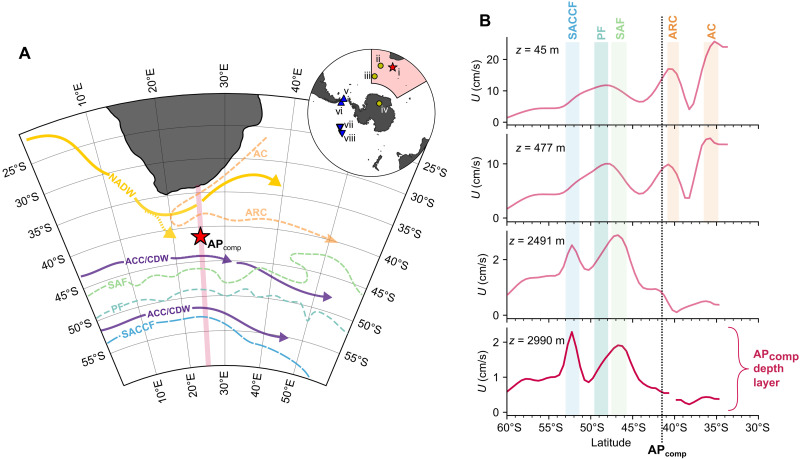
Circulation and major currents in the Agulhas Current region. (**A**) Circulation schematic of the Agulhas Current region. Solid arrows represent major pathways of deep water (CDW in purple; NADW in yellow); dashed orange arrows represent shallow subtropical circulation (AC, Agulhas Current; ARC, Agulhas Return Current); dashed green and blue lines represent major ACC jets/fronts as defined in (*92*). Map created with Cartopy in Python (https://pypi.org/project/Cartopy/). The inset map shows the location of key records relevant to this study: the AP_comp_ (i; this study), ODP 1090 (ii), ODP 1094 (iii), the Dome Fuji ice core (iv), PS97/085 (v), PS97/093 (vi), Site U1540 (vii), and Site U1541 (viii). (**B**) North-south flow speed profiles along a transect at 25°E [shown as a pink line in (A)]. Each panel represents a different depth level. The vertical black line shows the latitudinal position of the AP_comp_, and the shaded bars represent the location and depth of relevant currents in the region. Flow speed data are from the ECCOv4r3 climatology (*93*) (1992–2015) provided by the ECCO consortium (http://ecco-group.org/).

The Antarctic Circumpolar Current (ACC) is today the world’s largest ocean current, driving a continuous eastward transport of roughly 140 to 150 sverdrup (1 sverdrup, Sv = 10^6^ m^3^ s^−1^) of water around Antarctica (*1*). This circumpolar flow effectively isolates the Antarctic margins from warm surface waters of the subtropics and is an essential component driving meridional overturning circulation (*2*). The major flow of the ACC is concentrated in narrow jets that extend to the seafloor and are aligned with strong hydrographic fronts, divided (from north to south) into the Subantarctic Front (SAF), the Polar Front (PF), the Southern ACC Front (SACCF), and the Southern Boundary front (*3*) (Fig. 1). The ACC is delineated from the Subtropical Gyres (STG) to the north by the Northern Boundary. The strength and position of these jets and fronts are mainly determined by the Southern Hemisphere westerly wind (SHW) stress, which partially drives the ACC, as well as the temperature gradients across the fronts and the interaction between friction, bottom drag, and topography (*4*). This dynamical link implies that changes in the geometry and strength of the ACC could occur in response to shifting westerly wind patterns (*5*) and changes in buoyancy forcing (*6*). In addition, these changes in forcing may also alter the latitudinal extent of the STG (*5*, *6*), with important implications for interbasin exchange, such as via the Agulhas Leakage (*7*).

Toggweiler *et al.* (*8*) previously proposed a mechanistic link between the latitude of the SHW stress, the ACC, and the global carbon cycle, whereby an equatorward shift in the former under glacial boundary conditions corresponds to reduced upwelling of carbon-rich Circumpolar Deep Water (CDW) in the Southern Ocean (SO) and reduced North Atlantic Deep Water (NADW) formation in the North Atlantic. Accordingly, a poleward shift in SHW stress would instead invigorate CDW upwelling and NADW formation via enhanced northward Ekman transport across the latitude range of the Drake Passage and a more substantial return of warm, saline water through the Agulhas Leakage, respectively (*5*, *8*). Warming over recent decades has driven a poleward intensification of the SHWs (*9*), driving a corresponding response in the strength and meridional position of ACC jets and fronts (*10*–*13*). While eddy activity may act to diminish the sensitivity of ACC transport to westerly wind stress (*14*), this potentially constitutes a critical positive feedback with respect to future warming, as well as Pleistocene deglaciations and warm intervals. An equatorward SHW shift of several degrees has been proposed during Pleistocene glacial intervals (*15*, *16*), with subsequent poleward migrations invoked as central for invigorating CDW upwelling in the SO and driving the rapid rise in atmospheric carbon dioxide (CO_2_) across Late Pleistocene glacial terminations (*15*, *17*, *18*). Despite the widespread acceptance that the SHWs and ACC are key players in Pleistocene climate cycles, direct proxy evidence to test this theory remains sparse. For example, the most complete reconstruction of Pleistocene ACC flow currently available (*19*) is limited to sites poleward of 54°S, making it difficult to distinguish a weaker overall ACC [as proposed in (*19*) for glacial intervals] from an equatorward reorganization of ACC jets.

To address this, we present a high-resolution deep flow speed reconstruction from the northern edge of the ACC, spanning the past 1.9 Ma (1 Ma = 1 million years), with an average resolution of 1 kyr (1 kyr = 1000 years). The sediment core site, the Agulhas Plateau Composite (AP_comp;_ 41°S, 25°E, 3000-m water depth) (*20*), is located in the Subantarctic Zone (SAZ; here considered the region between the SAF and the Northern Boundary) south of Africa. We supplement this with reconstructions of the intermediate-deep ocean stable carbon isotope gradient (hereafter, chemocline) from co-registered samples during the key glacial-interglacial transitions associated with Marine Isotope Stage 5 (MIS 5) [from (*21*)], MIS 11, and MIS 31 (this study). The circulation in the region is dominated by the relatively shallow eastward-flowing Agulhas Return Current to the north and the deep-reaching (extending to the seafloor) ACC to the south (Fig. 1). Previous studies have shown that fluctuations in near-bottom flow speed (~3000-m depth) in this region are driven by changes in the proximity and/or strength of the ACC and associated SAF south of Africa (*22*). These ocean current dynamics are closely linked to deep water mass fluctuations; the ACC transports CDW, while the sluggish, geostrophic flow of NADW in the southeast Atlantic (*23*) is primarily transported into the southwest Indian Ocean as the Agulhas Undercurrent below the Agulhas Current (*24*). To reconstruct near-bottom flow speed variability, we measured the mean grain size of the noncohesive silt fraction [10 to 63 μm, known as “Sortable Silt” ($\bar{\text{SS}}$)] (*25*) in terrigenous sediments from the AP_comp_. This paleocurrent proxy ($\bar{\text{SS}}$) is highly sensitive to hydraulic sorting by bottom currents, with stronger flow corresponding linearly to higher $\bar{\text{SS}}$ (*26*). The slope of this relationship (flow speed versus $\bar{\text{SS}}$) is remarkably consistent across various deep-sea environments. However, the absolute speed (i.e., the *y* intercept of this relationship) is sensitive to the proximity of the sediment source (*26*, *27*). We therefore present our flow speed reconstructions as deviations from the Late Holocene average (relative change in flow speed, cm/s: Δ*U*_Holocene_; see Materials and Methods). To extend the existing AP_comp_ chemocline record (*21*), we measured the stable oxygen (δ^18^O) and carbon (δ^13^C) isotope composition of the deep-dwelling (~600-m water depth) planktic foraminifer *Globorotalia truncatulinoides* (sinistral) (this study) and the epibenthic foraminifer (~3000-m water depth) *Cibicides wuellerstorfi* (*20*) at high resolution across MIS 11 and MIS 31. The chemocline record in this region reflects the efficiency of nutrient utilization in the SO as well as the strength of vertical stratification and air-sea exchange (*21*, *28*). At glacial terminations, for example, intervals of enhanced CDW upwelling are reflected by the convergence of δ^13^C values between the deep and intermediate SO and a reduced overall biological pump efficiency (*21*, *29*).

## RESULTS

In the modern ocean, maximum SHW stress occurs around 55°S, aligning with the Drake Passage and driving strong zonal jets, facilitating upwelling through Ekman divergence (*2*). Recent work demonstrates that the SHW stress was shifted equatorward during the Last Glacial Maximum (LGM), no longer aligning with the Drake Passage (*15*). Under this scenario, model results predict an equivalent shift in the axes of the main ACC flow toward the equator, with flow speeds strengthening in the midlatitudes and weakening in high-latitude regions such as the Drake Passage (*30*, *31*). Should this glacial-interglacial mechanism hold, we might expect to see an antiphasing of ACC flow speeds, recorded by $\bar{\text{SS}}$ records, from the mid- and high-latitude ACC. Higher LGM flow speeds in the midlatitude SAZ have been previously inferred from the Agulhas Plateau region (*22*), central South Atlantic (*32*), and Southwest Pacific (*33*). Conversely, higher-latitude sites in the Drake Passage and central South Pacific broadly exhibit lower flow speeds during glacial MIS 2 and MIS 4 compared to the Holocene and last interglacial (*19*, *34*–*36*). Furthermore, in the South Atlantic and south of Africa, an equatorward shift or strengthening of the SAF during the LGM is supported by an increased supply of Patagonian and Antarctic sediment carried by the ACC, as evidenced by radiogenic isotopes (*37*) and more ACC-derived clay mineralogy [i.e., higher kaolinite/chlorite ratios (*38*)] in the midlatitude South Atlantic (*39*, *40*). In the southeast Indian Ocean (*41*, *42*) and Scotia Sea regions (*43*), the picture from published data is less clear, likely owing to the complex nature of the ACC jets and their interaction with sea ice and topographic steering. Nonetheless, our data show that at least in the Atlantic Sector and south of Africa, the ACC strengthened northward during the LGM, possibly in response to a northward migration in the SHWs of several degrees (*15*).

**Fig. 2.**
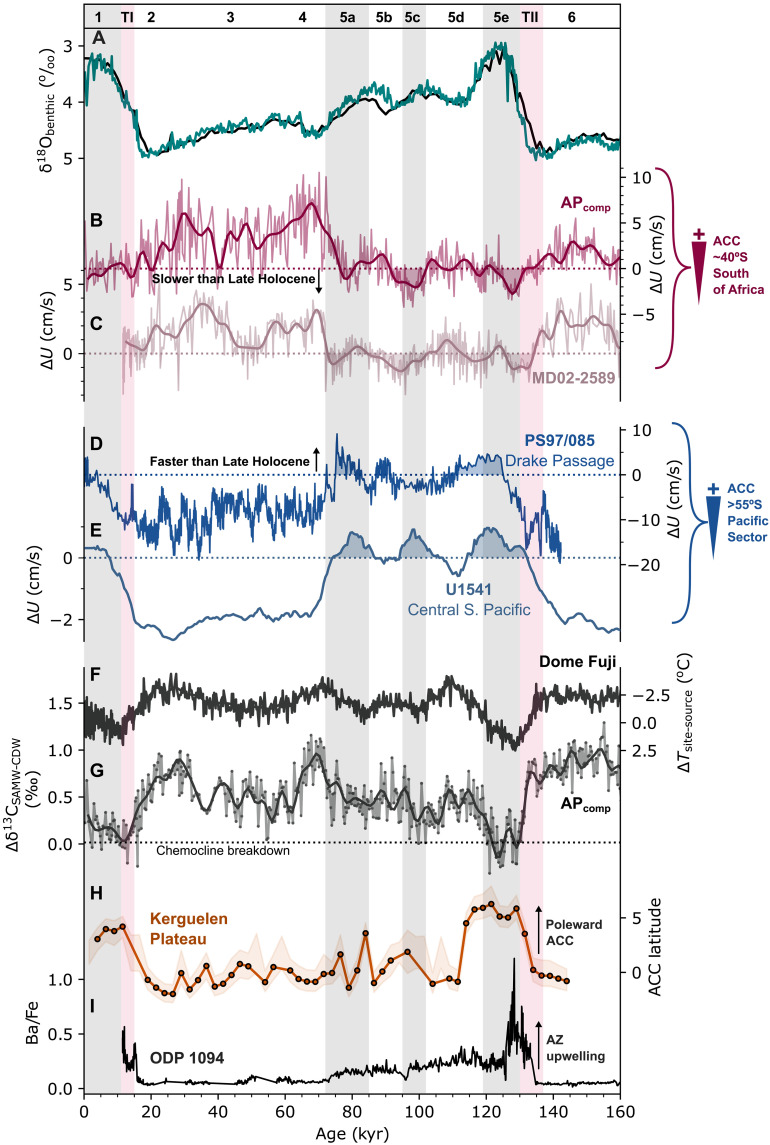
ACC reconstructions for the past 200 ka. (**A**) δ^18^O_benthic_ from the AP_comp_ [teal; (*20*)] and the updated global benthic δ^18^O stack [black; (*87*)]. (**B** and **C**) SAZ Δ*U*_Holocene_ from (B) the AP_comp_ (this study) and (C) MD02-2589 [southern Agulhas Plateau; (*22*)]. (**D**) Drake Passage Δ*U*_Holocene_ from PS97-085 [converted from $\bar{\text{SS}}$ + fine sand; (*36*)] and (**E**) South Pacific Δ*U*_Holocene_ from U1541 (*19*). (**F**) Source region–Dome Fuji ice core site temperature gradient (*49*). (**G**) AP_comp_ Δδ^13^C between *G. truncatulinoides* (sinistral) (mode water) and *C. wuellerstorfi* (CDW) (*21*). (**H**) Reconstructed ACC latitude (in degree migration from modern) in the Kerguelen Plateau region (*45*). (**I**) ODP 1094 Ba/Fe record, representing Antarctic Zone productivity (*51*).

Hydrographic reconstructions of the oceanic fronts of the ACC (*15*, *16*, *44*) support this interpretation. For example, a recent reconstruction of ACC latitude in the South Indian Ocean reveals a >4° poleward migration across Termination II (*45*), coincident with a rapid decrease in AP_comp_ flow speed. Geochemical reconstructions of density gradients reveal a minimal change (*46*) or a slight strengthening (*47*) of ACC transport during the LGM. However, it is important to note that substantial changes in relative flow across the ACC jet field and little change in total ACC transport may not be mutually exclusive (*48*). Conversely, some model results indicate that equatorward-shifted SHW stress results in weaker overall ACC transport (*31*). We suggest that a weaker yet northward-shifted ACC would satisfy the glacial scenario presented by $\bar{\text{SS}}$ data: (i) stronger flow in the SAZ south of Africa (where the ACC is essentially free from topographic constriction), (ii) weaker flow in the SAZ at the Kerguelen–St Paul Passage (*41*) and Drake Passage (*34*, *36*) (where the ACC is tightly bounded by shallow bathymetry to the north and south), (iii) weaker flow in the open Pacific Ocean south of ~54°S (*19*) as jets shift equatorward, and (iv) intensified Deep Western Boundary Current in the Southwest Pacific (*33*). The antiphase pattern in ACC flow speeds over the last glacial cycle between the midlatitude sites AP_comp_ (this study) and MD02-2589 (41°S, 25°E, 2660 m) (*22*) versus Drake Passage sites PS97/085 (58°S, 62°W, 3090 m) (*36*) and South Pacific International Ocean Discovery Program (IODP) site U1541 (54°S, 125°W, 3604 m) (*19*) is demonstrated in Fig. 2. These records show that ACC flow speed at midlatitude locations decreased markedly at the end of MIS 6 (~6-cm/s decrease across Termination II), with flow during the peak interglacial MIS 5e reaching ~3 cm/s lower than the Late Holocene. This deglacial decrease is coincident with increasing flow speeds at Drake Passage sites (Fig. 2; ~12-cm/s increase into MIS 5e), implying a poleward reorganization of the ACC during the transition from MIS 6 into MIS 5e.

**Fig. 3.**
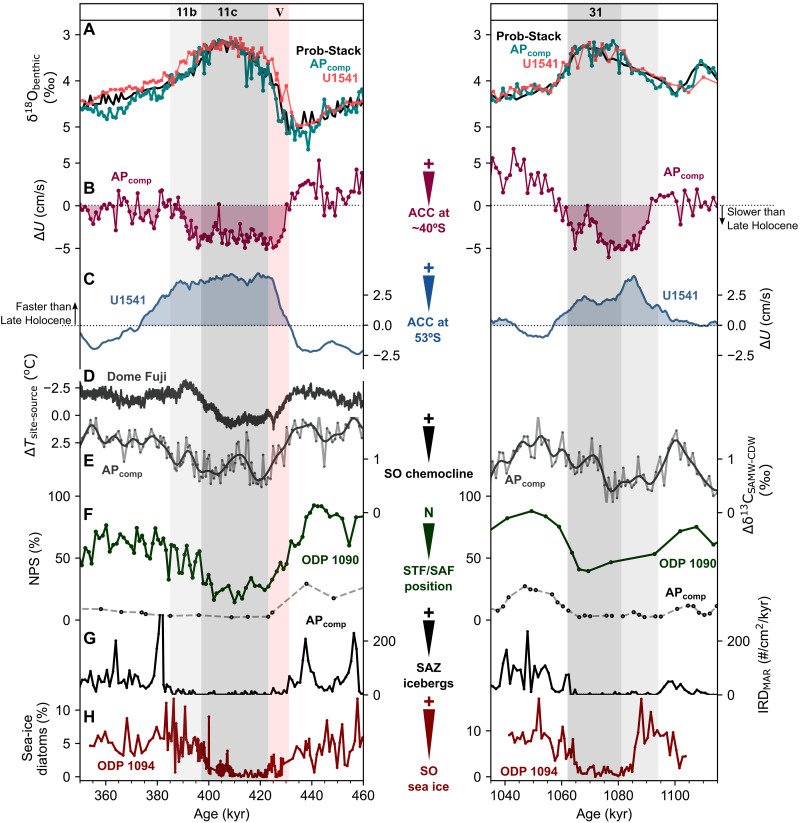
Reconstructed ACC dynamics across MIS 11 (left) and MIS 31 (right). (**A**) Benthic δ^18^O record from the AP_comp_ (*20*) (teal) and the updated global benthic δ^18^O stack (*87*). (**B**) AP_comp_ Δ*U*_Holocene_ (this study). (**C**) South Pacific Site U1541 Δ*U*_Holocene_ (*19*). (**D**) Source region–Dome Fuji ice core site temperature gradient (*49*). (**E**) AP_comp_ Δδ^13^C between *G. truncatulinoides* (sinistral) (mode water) and *C. wuellerstorfi* (CDW) (this study; the smoothed line shows a 7-kyr low-pass filter). (**F**) % *N. pachyderma* (NPS) abundance at ODP Site 1090 [dark green; (*56*)] and the AP_comp_ [black; (*58*)]. (**G**) Accumulation rate of ice-rafted debris at the AP_comp_ (*20*). (**H**) Percentage of sea-ice indicator diatom species at ODP Site 1094 [MIS 11 from (*67*); MIS 31 from (*68*)]. Peak interglacial MIS 11c is labeled, as is Termination V (V) and MIS 11b. The darker-shaded MIS 31 boundaries are given following the LR04 definition (*86*) with the earlier onset of MIS 31 following the Iberian Margin–derived definition (*94*) shown in lighter gray.

The zonal flow of the ACC and the SHWs is tightly linked to meridional overturning circulation and, hence, the upwelling of carbon-rich CDW in the SO (*2*). This physical-biogeochemical coupling can be observed clearly in the MIS 6/5 transition (Termination II; Fig. 2), whereby a poleward migration of the SHWs [as inferred from the Dome Fuji ∆T record (*49*)] coincides with a reduction in the strength or proximity of the SAF relative to the AP_comp_ (Fig. 2). Increased nutrient supply to the high-latitude Indian (*50*) and Atlantic sectors (*51*) and a breakdown in the SO chemocline (*21*) show that this coincided with an increase in wind-driven CDW upwelling in the SO (Fig. 2). To explore how ubiquitous this coupling is further back in time, we extend the AP_comp_ chemocline record to span two of the more well-studied “super-interglacials” of the Pleistocene: MIS 11 [~410 thousand years (ka)] and MIS 31 (~1070 ka) (Fig. 3). Both intervals are considered to be anomalously long and warm interglacials, with high sea surface temperatures observed in the SO (*52*, *53*) and high-latitude Northern Hemisphere (*54*); however, each occurred under distinct orbital forcing. A sequence of events similar to Termination II also occurred at the transition from MIS 12/11 (Termination V), wherein poleward-shifting SHWs (*49*) coincide with an abrupt decrease in AP_comp_ flow speed (>7 cm/s) and a concurrent increase in both Drake Passage and South Pacific flow speeds south of ~54°S (*19*, *55*), along with a near-collapse in the SO chemocline (Fig. 3). Micropaleontological reconstructions reveal coeval poleward migrations of SO fronts in the Atlantic (*56*) and Pacific (*57*) sectors of the SO, further indicated by a decline in the relative abundance of the subpolar-favoring planktic foraminifera *Neogloboquadrina pachyderma* (sinistral) at both the AP_comp_ (*58*) and Ocean Drilling Program (ODP) Site 1090 (*56*) (Fig. 3). Moreover, an enhancement of wind-driven CDW upwelling likely contributed to the abrupt rise in atmospheric CO_2_ (*59*) and would explain nutrient-rich conditions observed in the south Indian Ocean (*60*) across Termination V.

**Fig. 4.**
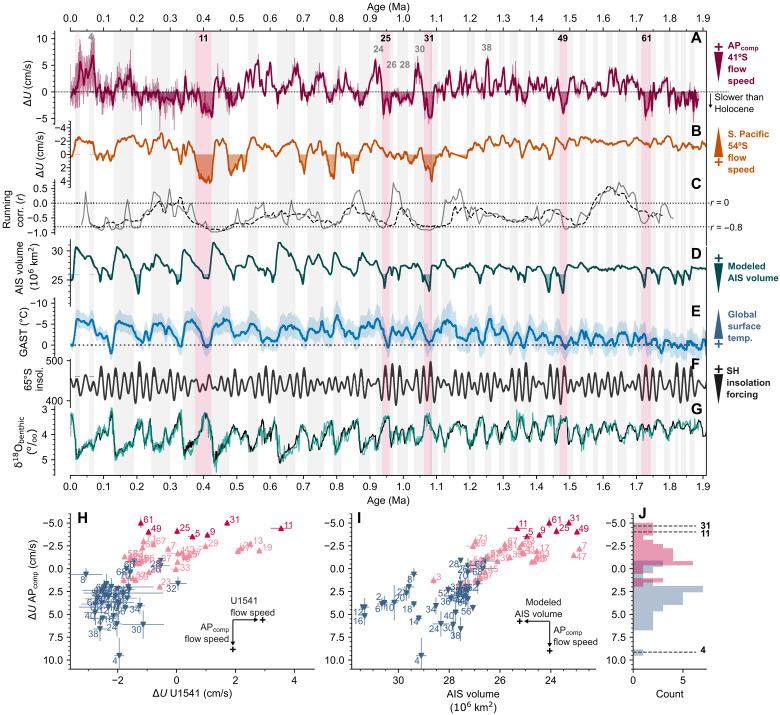
Interglacials and the ACC. (**A**) 1.9 Myr record of Δ*U*_Holocene_ from the AP_comp_ with a 7-kyr low-pass filter applied (dark red; top row). Times when flow is lower than the Late Holocene average are shaded in red. (**B**) Δ*U*_Holocene_ from Site U1541 in the central South Pacific (*19*). (**C**) Running Pearson correlation value between the AP_comp_ and Site U1541 Δ*U*_Holocene_ records (both records are downsampled with 6-kyr bins; 60- and 100-year window sizes represented by solid and dashed lines, respectively). (**D**) Modeled AIS volume (*75*). Values below 26 × 10^6^ km^3^ are shaded [West AIS collapse (*74*)]. (**E**) Global average surface temperature (GAST) estimated from proxy reconstructions (*73*). (**F**) January mean daily insolation at 65°S (*95*). Intervals with particularly strong insolation forcing are shaded. (**G**) Benthic δ^18^O record from the AP_comp_ (teal) and the updated global benthic δ^18^O stack (*87*) (black). (**H** and **I**) Cross-plots of MIS values (see online methods) for AP_comp_ Δ*U*_Holocene_ against AP_comp_ δ^18^O_benthic_ (H) and simulated AIS volume (*75*) (I) (blue marker, glacials; pink markers, interglacials). Error bars represent the spread of values determined for each MIS following bootstrap resampling of each time series (2σ, *n* = 1000). (**J**) Histogram of AP_comp_ Δ*U*_Holocene_ MIS values.

In addition to Terminations II and V, our data also reveal a weakening and/or poleward-shifted SAF south of Africa and subsequent collapse in the SO chemocline during the transition between MIS 32 and MIS 31 (Fig. 3). The MIS 32/31 transition is characterized by a rapid decrease in flow vigor at the deep Agulhas Plateau (~6 cm/s), with a corresponding increase in flow speed in the central South Pacific and Drake Passage (poleward of 54°S) (*19*, *55*). Micropaleontological evidence again supports a poleward shift in the SO fronts during MIS 31 (*56*, *58*, *61*), and although no ice core evidence for atmospheric circulation extends this far back, a minimum in wind-blown dust deposition in the Atlantic SAZ is consistent with a poleward shift in the main SHW band at this time (*62*). Last, each key interglacial interval highlighted here corresponds to the strongest negative correlations (sliding window Pearson’s *r*; Fig. 4) between the AP_comp_ and Site U1541 (*19*) flow speed records, likely indicative of meridional shifts in the ACC. Together, this multiproxy evidence demonstrates that the rapid reduction in near-bottom flow on the Agulhas Plateau represents a poleward reorganization of the ACC and is closely linked to enhanced CDW upwelling at the start of MIS 5, MIS 11, and MIS 31.

## DISCUSSION

Sea level estimates and ice-proximal geological evidence support a substantial ice retreat in at least some sectors of the Antarctic Ice Sheet (AIS) during peak interglacial MIS 5 (*63*), MIS 11 (*64*, *65*), and MIS 31 (*66*). During MIS 11 and MIS 31, anomalously warm sea surface temperatures in the open SO (*53*) and at ice marginal sites (*53*) as well as reduced sea-ice extent and iceberg survivability in the Atlantic SO (*20*, *67*–*69*) (Fig. 3) may indicate an ocean-driven mechanism for AIS retreat during these intervals. It is important to note that the potential saturation of sea-ice and ice-rafted debris proxies during warm periods means that further evidence is needed to determine whether sea-ice extent was lower during MIS 11 and MIS 31 compared to other, milder interglacials. A mechanistic link between global climate, AIS volume, and ACC flow south of Africa is further supported by analysis of the long-term maxima and minima in the AP_comp_ flow speed “intensity” for each MIS (see Materials and Methods). This “MIS analysis” reveals that among interglacials, the slowest flow speeds at the AP_comp_ occurred during MIS 11 (~410 ka), MIS 25 (~950 ka), MIS 31 (ca 1070 ka), MIS 49 (~1480 ka), and MIS 61 (~1730 ka) (Fig. 4). The highest flow speeds (among the glacial maxima) occurred during MIS 4 (~70 ka), MIS 24 (~921 ka), MIS 30 (~1037 ka), and MIS 38 (~1250 ka). Except for MIS 4, these glacials occurred during the Middle Pleistocene Transition (MPT; ~0.9 to 1.2 Ma), during which equatorward migrations of the PF (*70*), SAF (*71*), and STF (*58*) have been previously recorded in the Indian-Atlantic sector of the SO. It is perhaps puzzling to observe that the interim glacial intervals MIS 26 and MIS 28 are not linked to flow speed maxima, suggesting that orbital-scale shifts in the intensity and/or proximity of the northern ACC south of Africa were relatively muted during these intervals. Superimposed on this pattern of glacial and interglacial intensity are long-term mean shifts in AP_comp_ flow speed, characterized by a stepwise increase at ~0.9 Ma and a subsequent decrease at ~0.4 Ma (Fig. 4; also see fig. S6). These changes are consistent with the timing of PF migrations recorded by diatom mats in the South Atlantic sector of the SO (*70*), wherein fronts shifted equatorward at the MPT (~0.9 Ma) and returned poleward around the Mid-Brunhes Transition (~0.4 Ma). Coincident baseline shifts are also recorded in the deep flow speed variability at the SW Pacific ODP Site 1123, although opposite in sign. This may reflect key differences in the response of the ACC and the deep Pacific Inflow to climate forcing over the MPT and Mid-Brunhes. While significant negative correlations between flow speed records from the midlatitude AP_comp_ and the higher-latitude sites (AP_comp_ versus site U1541: *r* = −0.42, *P* < 10^−50^; AP_comp_ versus PS97/085: *r* = −0.39, *P* < 10^−10^) imply pervasive latitudinal shift ACC flow in the recent geological past, it is clear that to fully resolve changes in the strength and geometry of the entire ACC system, reconstructions would be required from a transect of well-dated sediment cores spanning the full suite of ACC fronts at a given longitude (an endeavor not possible with available existing SO sediment cores). Despite this, the agreement between our observations and climate model results (*30*, *31*, *48*) suggests that comparing the midlatitude AP_comp_ and high-latitude Drake Passage and South Pacific sites provides valuable insight into the reorganization of ACC flow on glacial-interglacial timescales.

With the multiproxy reconstructions available to us, we can however infer that the interglacial minima in AP_comp_ flow speeds during MIS 11, MIS 25, MIS 31, MIS 49, and MIS 61 likely represent poleward migration or weakening of the SAF jet and expansion of the STG south of Africa (*5*) (Fig. 4). From a water mass perspective, this equates to the ACC-driven CDW, which bathed the site during glacial intervals, being replaced by the relatively sluggish NADW underlying the Agulhas Current System (Fig. 1). This scenario is supported for MIS 31 by long-term minima in polar alkenone flux (*72*) and sea surface salinity (*71*) at ODP 1090, as well as a more “subtropical” nitrogen isotope composition of AP_comp_ foraminifera (*58*). Furthermore, these intervals coincide with comparable or higher-than-present global surface air temperatures (*73*) and substantially reduced AIS volume as indicated by long-term ice sheet simulations (*74*–*76*) (Fig. 4). Interglacial AP_comp_ flow speeds (MIS ensemble values) are strongly correlated with simulated interglacial AIS volume (*75*) (Pearson *r* = 0.66; Spearman’s rank ρ = 0.60); we also note that a significant correlation exists using alternative AIS simulations (*58*) (see Materials and Methods) and reconstruction of relative sea level (*77*) (*r* = −0.62; ρ = −0.53). Model experiments reveal that lowering the elevation of the West AIS results in decreasing (increasing) surface wind stress north (south) of 60°S due to reduced mechanical blocking and weaker katabatic winds, shifting the SHW jets poleward (*78*, *79*). As discussed above, meridional shifts in SHW stress can drive meridional reorganization of ACC flow, mainly where low Antarctic sea-ice extent maximizes the coupling between SHW strength and surface stress in the SO (*80*). Microfossil and ice-rafted debris evidence demonstrates that super-interglacials were characterized by prolonged reductions in sea-ice extent in the Atlantic sector of the SO (*20*, *69*) (Fig. 3). Alternatively, a poleward reorganization of ACC flow south of Africa may have relaxed the restriction of the Agulhas Leakage (*7*, *31*), weakening the recirculation of Indian Ocean waters and therefore reducing the interaction of mesoscale eddies between the Agulhas Return Current and the ACC in the region, although we note that the relatively shallow reach of the Agulhas Return Current (Fig. 1) precludes changes in the strength of the Agulhas Current System as a direct driver of observed flow speed changes at the AP_comp_. Moreover, strengthening the return flow of saline surface waters to the North Atlantic would likely promote NADW formation (*81*), which would result in a (transient) flushing of carbon from the deep ocean, possibly contributing to rising atmospheric CO_2_ concentrations during the early interglacial stages as the Atlantic Meridional Overturning Circulation deepens (*82*).

Prior to the MPT, each of the long-term flow speed minima (Fig. 4) aligns with minima in simulated AIS as well as maxima in Southern Hemisphere insolation forcing, corresponding to the eccentricity modulation of precession (at 100- and 400-kyr periods). Only after the MPT does this relationship become obscured, possibly reflecting the increasingly nonlinear response of Earth’s climate to orbital forcing as continental ice sheets enlarged (*83*). The breakdown in the seemingly predictable pre-MPT pattern outlined above is epitomized by MIS 11, wherein a super-interglacial occurred despite weak insolation forcing. The occurrence of a distinct reorganization of the ACC south of Africa, along with a breakdown in the SO chemocline and, thus, exhalation of SO-sequestered carbon at the onset of MIS 11, despite weak insolation forcing, emphasizes the importance of carbon cycle feedback in forcing SO conditions under warm climate states. Drawing an analogy to ongoing and future climate change, several studies have linked recent changes in the ACC system to a poleward trend in SHW stress (*10*–*13*). However, an increasingly important role for baroclinic changes linked to buoyancy forcing is also invoked (*6*, *84*, *85*). For example, relatively large (small) heat gain to the north (south) of the SAF over recent decades has resulted in a stronger meridional temperature gradient and thus accelerated upper-ocean flow along the northern edge of the ACC (*6*, *84*). Comparing this scenario with MIS 11 and MIS 31 reconstructions reveals a key difference with modern conditions. These paleo-super-interglacials were instead characterized by a weaker latitudinal temperature gradient [i.e., relatively more warming at high latitudes (*53*)]. While recent modeling studies highlight essential questions regarding the relative importance of wind and buoyancy forcing on trends in SO circulation, our data provide paleoclimate context for a proposed response of the ACC to a poleward trend in SHW stress (*10*). While the modern SO currently acts as the dominant oceanic sink of CO_2_ (*82*), our results show that projected changes in the SHWs and ACC could drive oceanic responses, which ultimately weaken the SO’s CO_2_ sink by enhancing the upwelling of “natural” carbon stored in the otherwise isolated CDW (*82*). Moreover, the long-term perspective offered by our data supports the proposed link between AIS retreat/collapse and poleward reorganization of the ACC, with important implications for the potential feedback that may be triggered by the projected retreat of the future AIS in response to anthropogenic climate change (*83*).

## MATERIALS AND METHODS

### Sediment cores and age models

All data presented in this study are from the AP_comp_, a stratigraphic framework presented in (*20*) consisting of proximal sediment core sites MD02-2588 (41°19.90′S, 25°49.7′E, water depth of 2907 m) and International Ocean Discovery Program Site U1475 (41°25.6′S, 25°15.6′E, water depth of 2669 m). The age model used here is the “LR04” timescale (*86*) for the AP_comp_, constructed in (*20*) by aligning benthic δ^18^O to the updated global δ^18^O benthic stack (*87*) (fig. S1). We also compare our data to previously published data from a sediment core in the Drake Passage region: PS97/093 (*55*). The age model used for PS97/093 was constructed in (*55*) by aligning the x-ray fluorescence Ca/Fe ratio to the benthic δ^18^O stack. We update this age model slightly for consistency with the AP_comp_ δ^18^O stratigraphy, although the resulting changes are minor (<5 kyr; fig. S1) and limited to adjusting the position of what we believe is the MIS 40 peak in Ca/Fe.

### Grain-size analysis

For the $\bar{\text{SS}}$ determination, grain-size analysis was performed on the terrigenous sediment fraction of AP_comp_ samples (*n* = 1955 stratigraphic samples). The terrigenous sediment fraction was isolated following the protocol of (*25*) by removing calcium carbonate (CaCO_3_) (two overnight leaches with 2 M acetic acid) and biogenic opal [5 hours of digestion at 85°C in sodium carbonate (Na_2_CO_3_)] before rinsing with deionized water and dispersing in sodium hexametaphosphate. Prior to analysis, samples were placed on a rotating wheel for > 24 hours and ultrasonicated for 3 min. Grain-size analysis was performed for MD02-2588 and Site U1475 using a Beckman Multisizer III Coulter Counter and Beckman Multisizer IV Coulter Counter, respectively, at Cardiff University. Samples were analyzed ≥ 2 times until $\bar{\text{SS}}$ (defined as the geometric mean of the 10- to 63-μm size range) converged to within 0.3 μm. The final $\bar{\text{SS}}$ value is reported as the average of these runs. If the intrasample SD was high (>0.5 μm), then the data were dismissed and the sample remeasured.

To quantitatively compare changes in near-bottom flow speed between sites discussed in this study, we convert all $\bar{\text{SS}}$ records first to anomalies from Holocene average and then from grain size (micrometers) to flow speed (centimeters per second). For each $\bar{\text{SS}}$ record discussed and plotted, we take the Holocene average to be the average $\bar{\text{SS}}$ value for the 0- to 7-kyr interval (or, where no 0-kyr data are available, the uppermost 7 kyr of each record). No data are available for ODP Site 1123 (*33*) and MD02-2589 (*22*) after 7 kyr ago, so we take the 12- to 7-kyr interval average. For the conversion from $\bar{\text{SS}}$ to flow speed, multiple calibration lines exist. Here, we apply the “main-line” equation from (*26*) to sites outside of the Drake Passage. Sites within the Drake Passage region are calibrated using the regional equation from (*88*). The main-line equation gives a sensitivity of 1.26 ± 0.18 cm s^−1^ μm^−1^ (±2 SD) for Coulter counter data and 1.36 ± 0.19 cm s^−1^ μm^−1^ for Sedigraph data. Moreover, the main-line equation yields a similar sensitivity to the equation determined by the flume tank experiments in (*27*); applying either calibration has little impact on the resulting flow speed reconstructions. A comparison of the $\bar{\text{SS}}$ and Δ*U*_Holocene_ records is shown for various calibration equations in fig. S4.

### Carbon isotope measurements

Stable isotope (δ^13^C) measurements were made on deep-dwelling planktic foraminifera *G. truncatulinoides* (sinistral) specimens identified after dry-sieving each sample to isolate the 250- to 315-μm size fraction. The number of specimens measured in each sample (hereafter *n*) depends on the availability of morphologically distinct and well-preserved *G. truncatulinoides* shells; the target number was 15. However, *n* ranged from 3 to 15. For isotope analyses of large samples (*n* > 7), 100 to 250 μg of crushed sample was weighed using a Sartorius microbalance (precision, ±1 μg) and measured on a Finnigan Delta V Advantage coupled online with a Gasbench II. Long-term external precision is < 0.06‰ for δ^13^C, and internal SDs are generally < 0.1‰. Smaller samples (*n* ≤ 7) were measured using a Thermo Finnigan MAT 253 mass spectrometer coupled online to a Carbo Kiel carbonate preparation device with a long-term precision of < 0.05‰ for δ^13^C. All results are calibrated to an internal laboratory standard (BCT63) and reported relative to the Vienna Pee Dee Belemnite scale. All measurements were made at Cardiff University.

### Marine isotope stage analysis: Maximum intensity

To compare conditions among different MISs, we derive a single representative value for each stage from relevant datasets (table S2). One option is to average the data across the specified interval (i.e., the upper and lower age boundaries for each MIS according to some predetermined MIS definition). However, this approach relies on the assumption that each MIS constitutes a single stationary value, representative of the entire glacial or interglacial in question (*89*), while also dampening the values for each stage by including transitional data points between intervals. A second approach selects a single data point for a single point in time within each MIS, defined, for example, by the interglacial insolation peak or glacial ice volume maximum (*90*). This approach, while effective for selecting a time slice to simulate, is less helpful in constructing an ensemble of various proxy values for each MIS because leads, lags, and age model uncertainties may result in asynchronous expression of glacial or interglacial conditions between different regions and proxy systems (i.e., the maximum $\bar{\text{SS}}$ value within glacial stages generally precedes the peak in δ^18^O). In this study, we follow (*91*) in finding the maximum/minimum proxy value within each MIS interval. This requires the a priori assumption that each time series consistently exhibits a glacial-interglacial pattern, with maximum or minimum values faithfully representing the intensity of each stage, in other words, taking the highest (or lowest) value for each MIS, regardless of where within the given MIS it occurred. To compare records with varying temporal resolutions, each dataset is low-pass filtered to remove high-frequency variability (>1/5 kyr). For $\bar{\text{SS}}$, the maximum value is taken for glacials and the minimum value for interglacials, following the observed pattern of high (low) glacial (interglacial) values. Last, because the datasets in question are either tuned to or derived from the global benthic δ^18^O stack, we use the LR04 (*86*) definitions for the bounds of each MIS, taking even stages to be glacial and odd stages to be interglacial (sensu lato). Filtered records and the estimated MIS values are given in fig. S7 and reported in table S2.

### Statistical analysis of the MIS ensemble table

Pearson’s *r* and Spearman’s rank ρ correlation coefficients between interglacial values for each of the variables included in our MIS ensemble table are shown in fig. S8. For interglacials, AP_comp_ flow speed is most strongly correlated with simulated AIS volume (*75*), yielding a Pearson correlation coefficient of *r* = 0.66 ± 0.15 and a Spearman’s rank correlation ρ of 0.61 ± 0.20. The following strongest correlations are with reconstructed relative sea level (*77*) (*r* = −0.62; ρ = −0.53), AP_comp_ δ^18^O_benthic_ (*20*) (*r* = 0.52; ρ = 0.40), and global surface air temperature (*73*) (*r* = −0.47; ρ = −0.41). We note that the strong correlation between AP_comp_ flow speed and simulated AIS volume is consistent when the ANICE model results (*75*) are substituted for the ensemble simulations in (*76*) (fig. S9).
