# Supplementary material for: Shifting Antarctic Circumpolar Current south of Africa over the past 1.9 million years
Source: Sci Adv. 2025 Jan 1;11(1):eadp1692. doi: 10.1126/sciadv.adp1692 (PMC11691690; doi:10.1126/sciadv.adp1692)
Supplement: Supplementary file 1 — Supplementary Text Figs. S1 to S12 Tables S1 and S2 [file sciadv.adp1692_sm.pdf]

Science Advances
AAAS

Supplementary Materials for

Shifting Antarctic Circumpolar Current south of Africa over the past 1.9 million years

Aidan Starr *et al.*

Corresponding author: Aidan Starr, aidan.starr@climate-x.com; Ian R. Hall, hall@cardiff.ac.uk

Sci. Adv. **11**, eadp1692 (2025)
DOI: 10.1126/sciadv.adp1692

This PDF file includes:

Supplementary Text
Figs. S1 to S12
Tables S1 and S2

Supplementary Text

IODP Site U1475 – MD02-2588 Continuity

To present a continuous record of near-bottom flow speed through the Early to Late Pleistocene, $\overline{SS}$ is measured on both Site U1475 and MD02-2588, presented as a continuous stratigraphic series (the AP_{comp} ; Supplementary Fig. 2). The two sites are geographically close (within 0.2º of latitude and 0.6º of longitude of each other) and are separated by around 250m water depth. The difference in depth as well as topographic features such as sediment waves and mounds on the southern Agulhas Plateau may result in hydrodynamic differences between the two sites. Although only covering a single glacial-interglacial transition, data across the overlapping sections of both sites reveal an offset in $\overline{SS}$: Site U1475 (the shallower site) is ~2µm higher than MD02-2588, equating to a vertical flow speed gradient of ~1.4 cm/s per 100 m according to the calibration of (*26*). In order to make the Site U1475 $\overline{SS}$ data consistent with the MD02-2588 $\overline{SS}$ data, we apply a –2µm offset to the former. This allows us to estimate the Holocene anomaly (see below) for the full 1.9 myr interval and allows us to convert $\Delta\overline{SS}_{Holocene}$ to $\Delta\ U_{Holocene}$ (flow speed anomaly in cm/s) following the "main line" equation of *(26).* (see below for elaboration on calibration choice). The result of applying this offset has only a minor effect on the overall shape and low frequency characteristic of the AP_{comp} record overall. This is demonstrated in Supplementary Fig. 3, showing a comparison between the raw data (Supplementary Fig. 3a), the offset-applied data (Supplementary Fig. 3b), a 'unit-variance' approach whereby the two constituent records are standardised to mean = 0 and standard deviation = 1 before combining (Supplementary Fig. 3c), and the Holocene anomaly of the offset-applied data (Supplementary Fig. 3d).

Utility and considerations for the $\overline{SS}$ proxy at the Agulhas Plateau

The utility of $\overline{SS}$ to reconstruct near-bottom flow speed (and hence ACC proximity and/or strength) at the Agulhas Plateau relies on the assumption that hydraulic sorting is the dominant control on grain size in the 10-63µm 'Sortable Silt' range. More accurately, it depends on the dominance of selective deposition in the sorting of this size fraction (25). Encouragingly, seismic data provide evidence for a sedimentary environment which interacts extensively with current patterns on the Agulhas Plateau (110). Moreover, the Agulhas Plateau is bathymetrically isolated from the African continent, ascending ~2500m above the surrounding deep basins, minimising the potential influence of down-slope and continental shelf processes which might complicate the application of $\overline{SS}$. However, grain size through time can be influenced by changes in the supply of sediment, and any attempt to directly relate $\overline{SS}$ to absolute flow speed must also consider the effect of 'downstream fining' along a flow path (106). Furthermore, suspicion has been cast as to whether sediments containing ice-rafted debris (IRD) deposits are sufficiently current-sorted for the application of paleo-flow speed proxies (102). The presence of IRD in Pleistocene AP_{comp} sediments (20) means that a potential relationship must be considered here. Following ref 105, sediment will be current-sorted regardless of its origin (ice-rafted or otherwise) provided that flow speeds remain relatively high and the deposition of IRD is neither too high nor abrupt. Furthermore, in the Drake Passage region, where IRD deposition is substantially higher than at the Agulhas Plateau due to the proximity to Antarctica, no correlation between IRD and $\overline{SS}$ is evident, nor is an influence of IRD peaks on the correlation between $\overline{SS}$ and SS% (36), one proposed test for an IRD influence on $\overline{SS}$. Finally, there is only a weak correlation between IRD concentration and $\overline{SS}$ at the AP_{comp} (Pearson r^2 = 0.24) despite a relationship between the two proxies likely being exaggerated by complementary environmental

conditions favouring both IRD deposition and higher flow speed, such as a northward expansion of the ACC with the associated SO fronts.

Furthermore, the potential for variations in sediment source to affect Sortable Silt means that sea levels fluctuations must also be considered, owing to the exposure and export of terrigenous sediment from the South African margin. However, reconstructions of shelf exposure and sediment export from the South African margin (98) deviate substantially from the pattern of glacial (interglacial) Sortable Silt maxima (minima).

Quantitative flow speed reconstructions from $\overline{SS}$ records

In order to quantitively compare changes in near-bottom flow speed between sites discussed in this study, we convert all $\overline{SS}$ records first to anomalies from Holocene average, and then from grain size (µm) to flow speed (cm/s). For each $\overline{SS}$ record discussed and plotted, we take the Holocene average to be the average $\overline{SS}$ value for the 0 – 7 kyr interval (or, where no 0 kyr data is available, the uppermost 7 kyr of each record). For ODP Site 1123 (33) and MD02-2589 (22), no data is available after 7 kyr ago and so we take the 12 – 7 kyr interval average. For the conversion from $\overline{SS}$ to flow speed, multiple calibration lines exist. In this study, we apply the "main line" equation from (26) to sites outside of the Drake Passage. Sites within the Drake Passage region are calibrated using the regional equation from ref (88). The "main-line" equation gives a sensitivity of 1.26 ± 0.18 cm s^{-1}/µm (±2s.d.) for Coulter Counter data and 1.36 ± 0.19 cm s^{-1}/µm for Sedigraph data. Moreover, the "main line" equation yields a similar sensitivity to the equation determined by the flume tank experiments of (27); applying either calibration has little impact on the resulting flow speed reconstructions. Comparison of $\overline{SS}$ and $\Delta\ U_{Holocene}$ are shown for various calibration equations in Supplementary Fig. 4.

Drivers of deep flow dynamics at the Agulhas Plateau composite

The southern Agulhas Plateau is situated in a complex hydrographic and dynamical region. Shallow circulation in the region is dominated by the Agulhas Current, Agulhas Retroflection, and Agulhas Return Current (ARC), which constitute the western extension of the Indian Ocean subtropical gyre (96). This subtropical current system is bounded to the south by the equatorward edge of the ACC; specifically the Subantarctic Zone which constitutes the net eastward flowing waters between the SAF and Northern Boundary of the ACC. At Agulhas Plateau longitudes, the SAF and NB are located ~45ºS and ~39ºS, respectively (92). Deep hydrography on the southern AP is dominated by the competing influence of NADW, spreading south-eastward on its exit from the South Atlantic, and CDW transported at depth by the deep reaching ACC. It is this balance between the geostrophic, relatively sluggish NADW and the wind-driven, relatively vigorous CDW that ultimately influences the dynamical conditions on the southern Agulhas Plateau on Glacial-Interglacial timescales (22). For example, the ARC is unlikely to interact with the seafloor of the southern Agulhas Plateau (the ARC crosses the 3000m isobath without deflection over the northern Agulhas Plateau (101)). Moreover, the modern position of the first ARC meander draws the core of the ARC well to the north of the AP_{comp} site, and this meander is remarkably stationary, with little shift over a 15-year observational time period (during which time <0.1% of tracked particles passed over the AP_{comp} site (96), nor over longer geological intervals as demonstrated by detrital Sr isotope reconstructions (100) and seismic evidence from the northern Agulhas Plateau (110). Figure 1b demonstrates the ARC has little influence on deep flow at <2500m, and that the deep-reaching SAF jet extends to the seafloor at least at 2990m water depth. It follows that on paleoclimate

timescales, changes in near-bottom flow speed at the AP_{comp} are predominantly driven by changes in the proximity and strength of the ACC (in particular the nearby SAF). This is supported by the good correspondence between AP_{comp} flow speeds and: (1) the portion of "ACC" derived clay in sediment cores from the Agulhas Plateau and Cape Basin regions (Supplementary Fig. 11); (2) the percentage of polar water alkenones at ODP Site 1090, near the modern NB (Supplementary Fig. 12); (3) the percentage of subpolar-dwelling foraminifera species *N. pachyderma* at both the AP_{comp} and ODP Site 1090 (Supplementary Fig. 12). For example, over the last glacial cycle, higher AP_{comp} flow speeds during MIS 2 and 4 coincide with colder SSTs, as well as increased opal and carbonate productivity (Supplementary Fig. 11); in other words, more nutrient-rich subpolar conditions associated with an equatorward migration of the SAF. The antiphase relationship between the AP_{comp} and Drake Passage/South Pacific flow speed reconstruction is demonstrated by negative Pearson *r*: AP_{comp} vs PS097/085 (0 – 142 ka) = –0.39 ± 0.08 (2.5% bootstrap confidence level); AP_{comp} vs PS097/093 (0 – 1360 ka) = – 0.24 ± 0.06 (2.5% bootstrap confidence level); AP_{comp} vs Site U1541 (0 – 1850 ka) = – 0.42 ± 0.04 (2.5% bootstrap confidence level); and AP_{comp} vs Site U1540 (0 – 1850 ka) = – 0.42 ± 0.04 (2.5% bootstrap confidence level). Results of bootstrap correlation coefficients are shown in Supplementary Fig. 10.

Long-term trend and change-point detection

In order to determine long-term trends and step-changes in AP_{comp} $\Delta U_{Holocene}$, a range of offline Change-Point Detection (CPD) algorithms are applied to the standardized data after first interpolating to a linear age scale (Δt = maximum spacing between raw data points). CPD is a signal-processing technique which considers the time series to be 'piecewise non-stationary', meaning some characteristic changes at one or more unknown points in time. The four algorithms used here are a Pruned Exact Linear Time (PELT) search, a Dynamic Programming search, Binary Segmentation search, and Window-Based Search (see 109) for a detailed description of the algorithms employed here), though we note that the result of no single approach is taken to be absolute. Instead, transitions that are identified by multiple approaches and could also be identified by visual inspection are used to approximate segments in the time series. As shown in Supplementary Fig. 6, multiple change-points can be assigned to the time-series, and the CPD algorithm results are not always unanimous. 3 of the 4 algorithms detect a change-point between 400 and 500 ka, which we assign to 430 ka based on a peak in rolling-standard deviation. This transition displays a sharp decrease in flow speeds from MIS 11 into MIS 10. In addition, 3 of the 4 methods identify two transitions aligning roughly with the Mid-Pleistocene Transition (MPT); one at 930 ka (MIS 25/24) and another at around 1060 ka (MIS 31/30). These are characterised by increased glacial-interglacial variance separating lower (higher) frequency glacial cycles after (before) the MPT. Between these transitions, mean values are low with supressed glacial-interglacial variability. Additional change-points are identified in the >1060 ka section, however, these are less clear by eye and less obvious in the rolling standard deviation, and no change-point are formally defined before the MPT. The long-term AP_{comp} flow speed $\Delta U_{Holocene}$) mean increases slightly between the pre-1060 ka interval (0.17 cm/s, 1σ = 2.22cm/s) and post-930 ka interval (0.92 cm/s, 1σ = 2.12 cm/s) before decreasing into the post-430 ka interval (0.08 cm/s, 1σ = 2.59 cm/s); each of these three means are significantly different according to 2-sided t-tests.

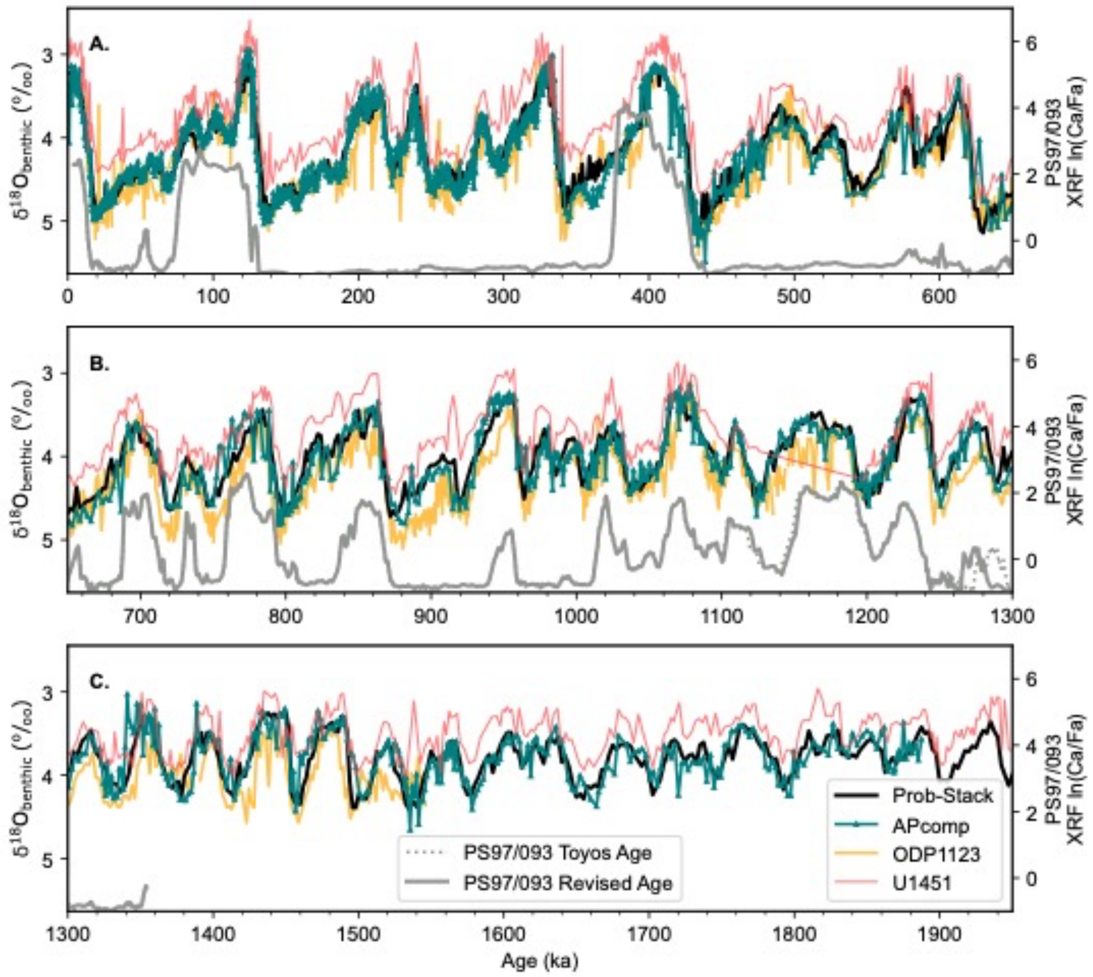

Fig. S1. Primary age-depth models used in this study.

Solid black line is the 'Probabilistic benthic stack' (87) (the tuning target for this study). The teal curve is the $\delta^{18}O_{benthic}$ record from the AP_{comp} (20) orange is the $\delta^{18}O_{benthic}$ record from ODP Site 1123 (99), and pink is the $\delta^{18}O_{benthic}$ record from IODP Site U1541 (107). Grey curves are the XRF Ca/Fe record from PS97/093 (55); the dashed grey line is the updated chronology used in this study.

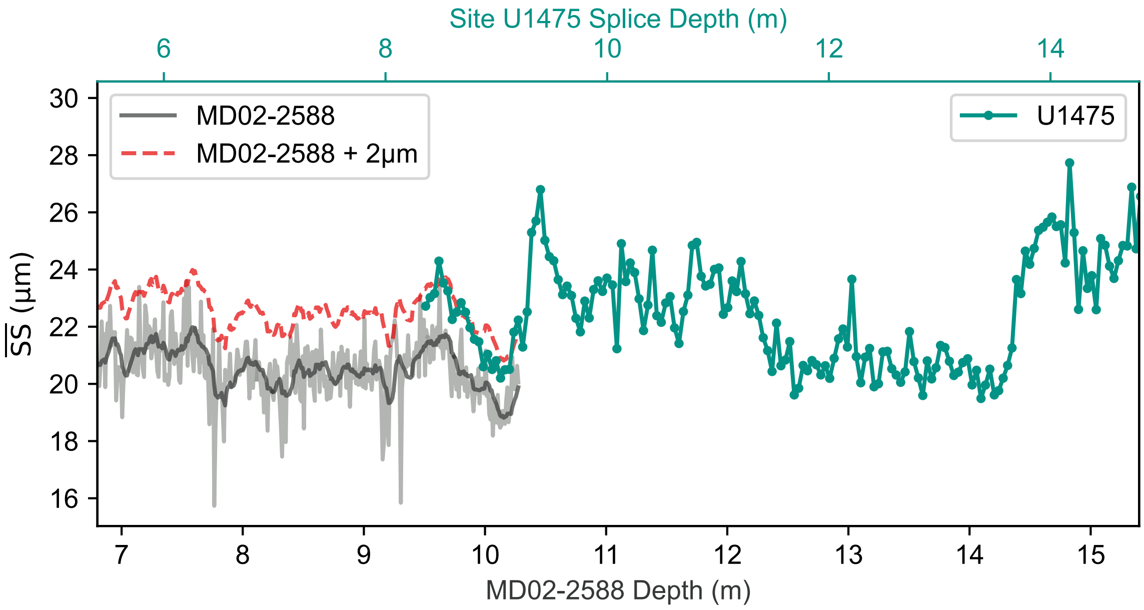

Fig. S2. Overlap between MD02-2588 and Site U1475.

$\overline{SS}$ data showing the splice-point between the constituent records of the AP_{comp}. The red dashed line shows the MD02-2588 data with a 2µm offset applied to match the Site U1475 data.

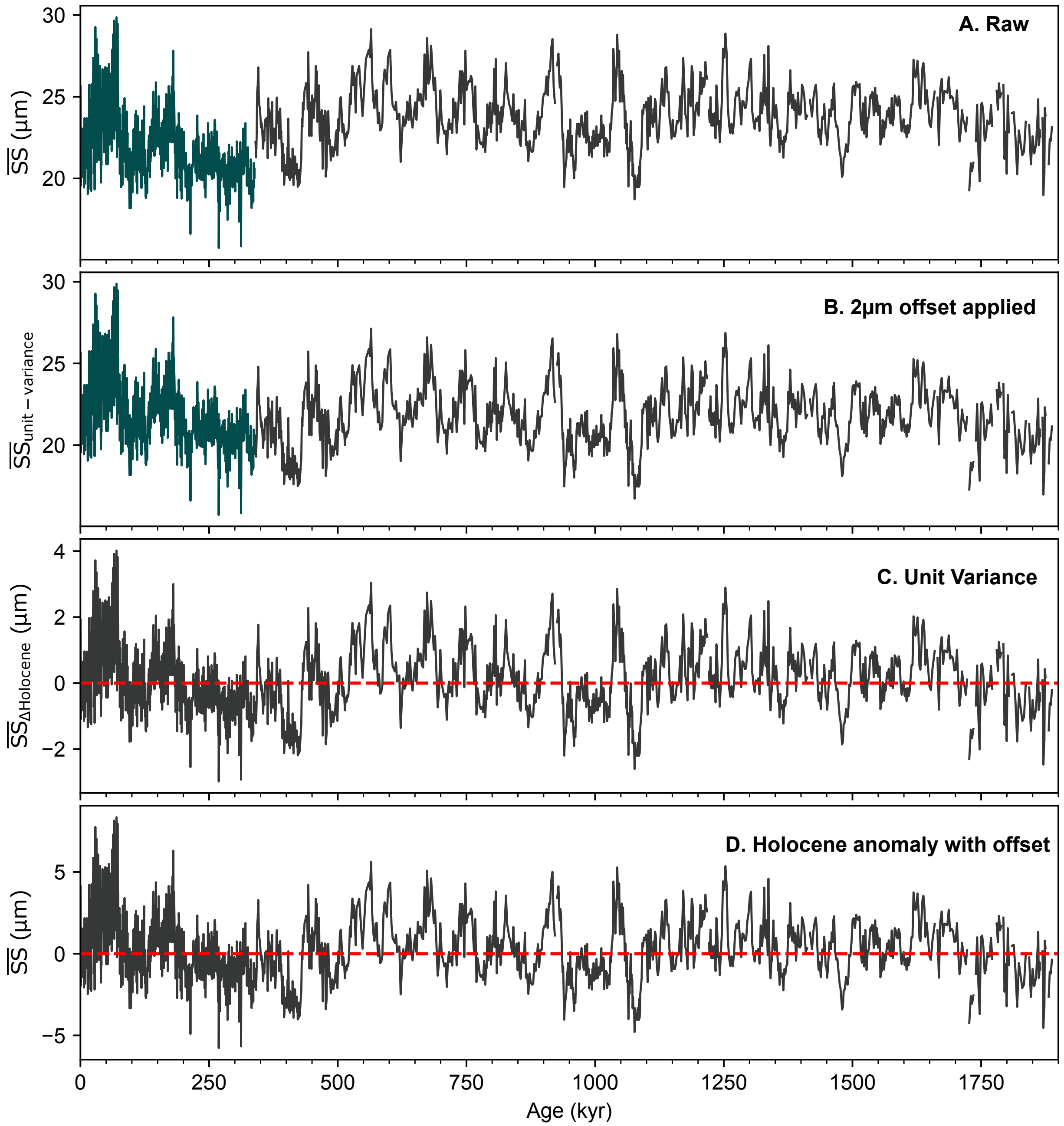

Fig. S3. Record of $\overline{SS}$ continuity between MD02-2588 and Site U1475. Green curves are the MD02-2588 part of the splice and grey are the Site U1475 component. Panel (a) is the raw $\overline{SS}$ data from both sites, uncorrected. Panel (b) shows the continuity after applying a 2μm offset. Panel (c) shows the continuity after first converting both records to their respective unit variance (mean 0; standard deviation 1). Panel (d) shows the Holocene $\overline{SS}$ anomaly after applying the 2μm offset.

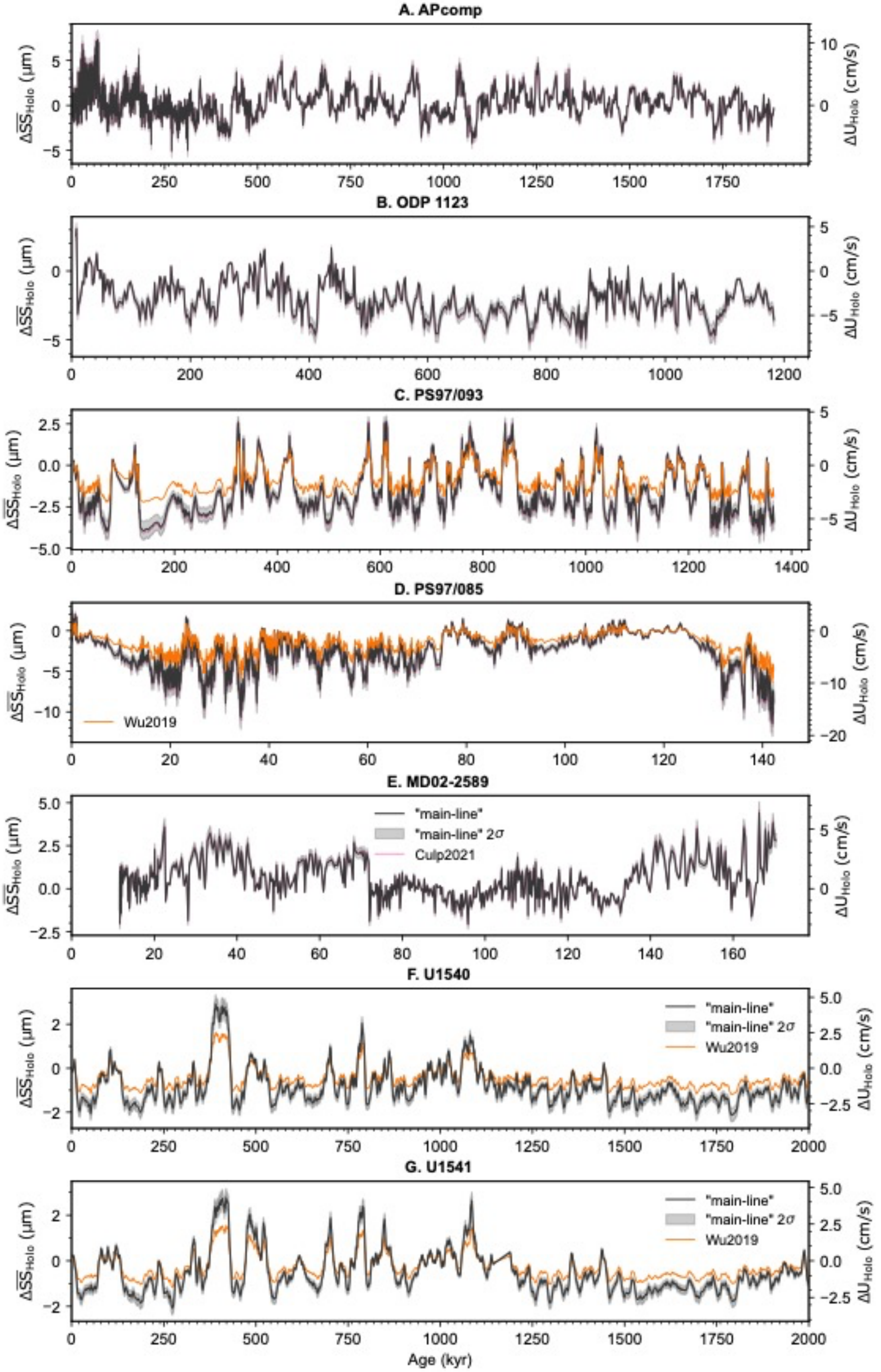

A. APcomp
B. ODP 1123
C. PS97/093
D. PS97/085
Wu2019
E. MD02-2589
"main-line"
"main-line" 2σ
Culp2021
F. U1540
"main-line"
"main-line" 2σ
Wu2019
G. U1541
"main-line"
"main-line" 2σ
Wu2019
ΔSS_Holo (μm)
ΔU_Holo (cm/s)
Age (kyr)

Fig. S4. Conversion from $\overline{SS}$ to $\Delta U_{Holocene}$ for records used in this study. Each panel shows the conversion from $\overline{SS}$ (in μm) to $\Delta U_{Holocene}$ using the 'main line' calibration (26) (grey line; shading gives 2σ uncertainty) and the laboratory-derived calibration (27) (red line). The calibration from ref (88), is also shown for the two Drake Passage records (PS97/093 and PS97/085).

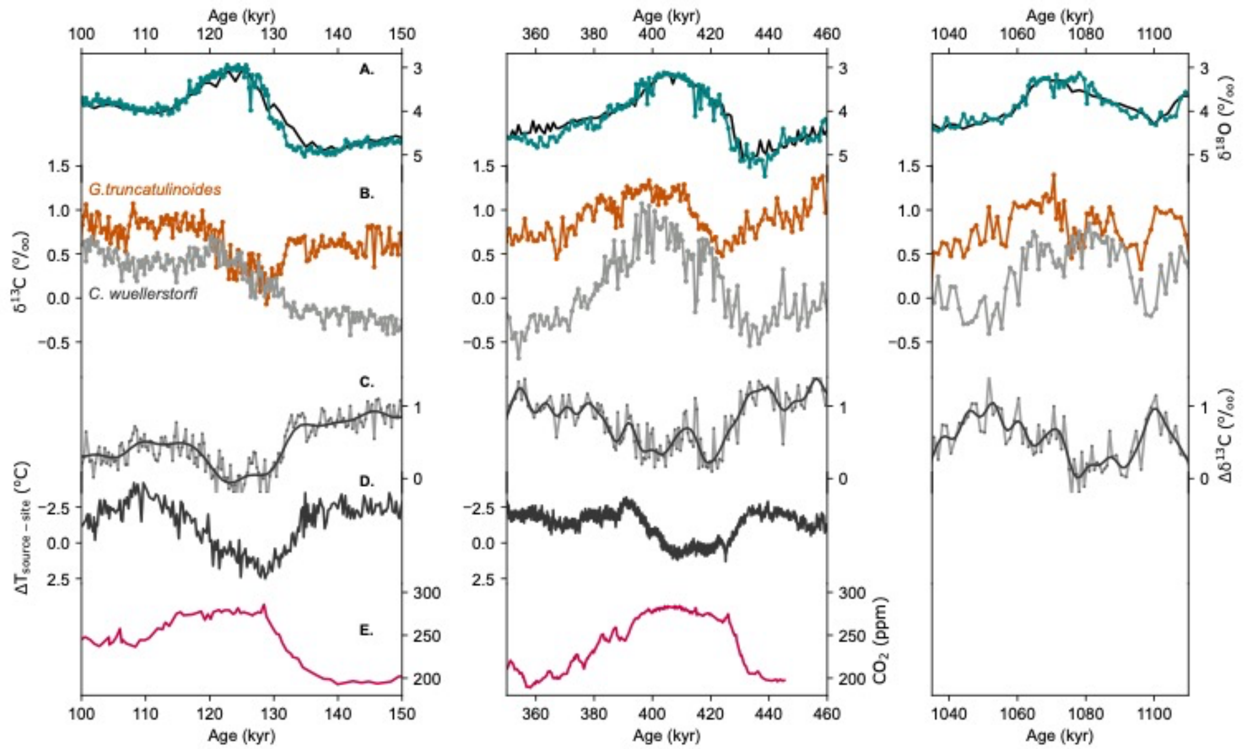

Fig. S5. Carbon isotope gradient record for the AP_{comp}. Top row: $\delta^{18}O_{benthic}$ from the AP_{comp} (teal) and the updated global benthic $\delta^{18}O$ stack (black; 87). Upper middle: $\delta^{13}C$ of *G. truncatulinoides sinistral* (orange) and *C. wuellerstorfi* (grey); left panel is data from ref (21) centre and right panels are new data from this study. Middle row: the $\Delta\delta^{13}C$ gradient between *G. truncatulinoides* and *C. wuellerstorfi* from the upper middle row. Solid black line shows a 1/5 kyr low pass filter. Lower middle row: Source region – Dome Fuji ice core site temperature gradient (49). Bottom row: Ice core reconstructions of atmospheric CO_2 concentration (104).

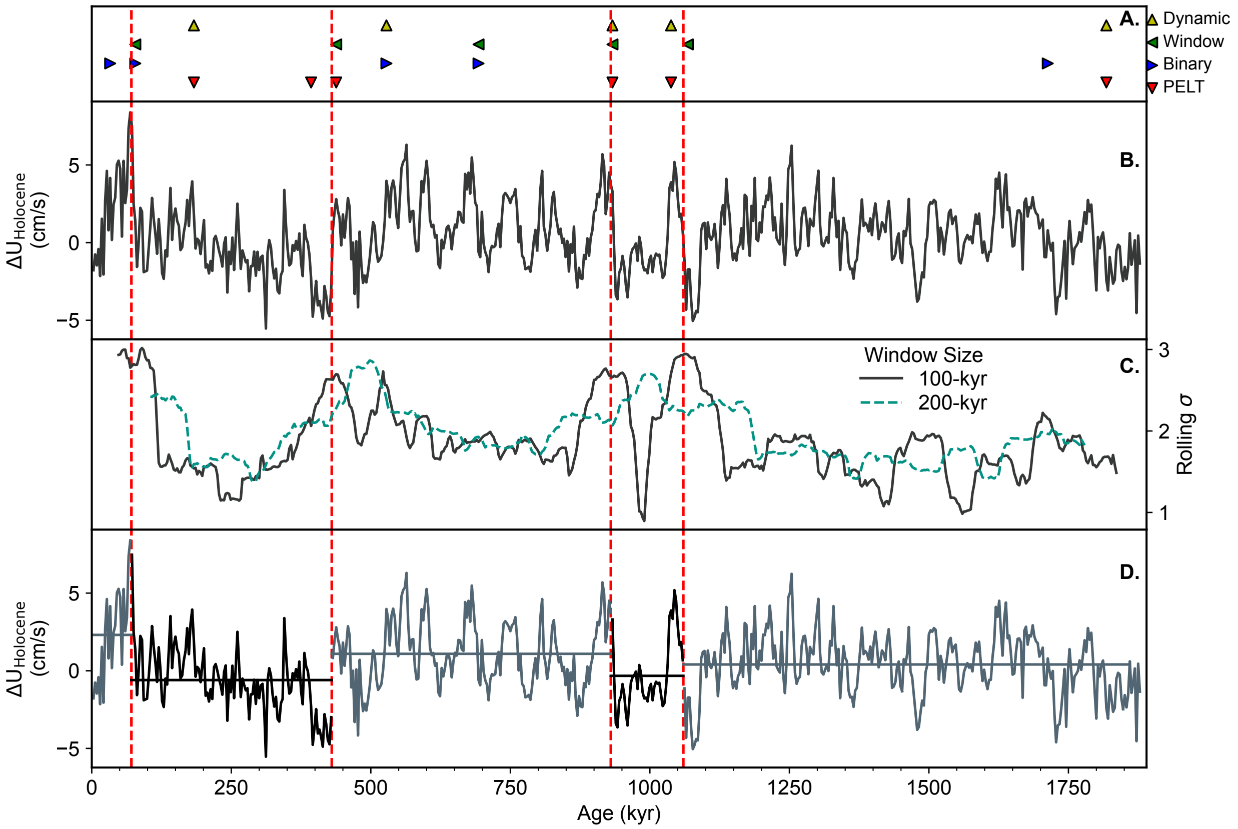

Fig. S6. Change Point Detection. (a) Transitions detected by the 4 algorithms described in the online methods. The AP_{comp} $\Delta U_{Holocene}$ record (b) and its rolling-window standard deviation (σ; c) are also shown. The bottom panel (d) shows AP_{comp} $\Delta U_{Holocene}$ segmented at the transitions denoted by vertical red dashed lines, as discussed in the Online Methods. Solid horizontal lines represent the segment average.

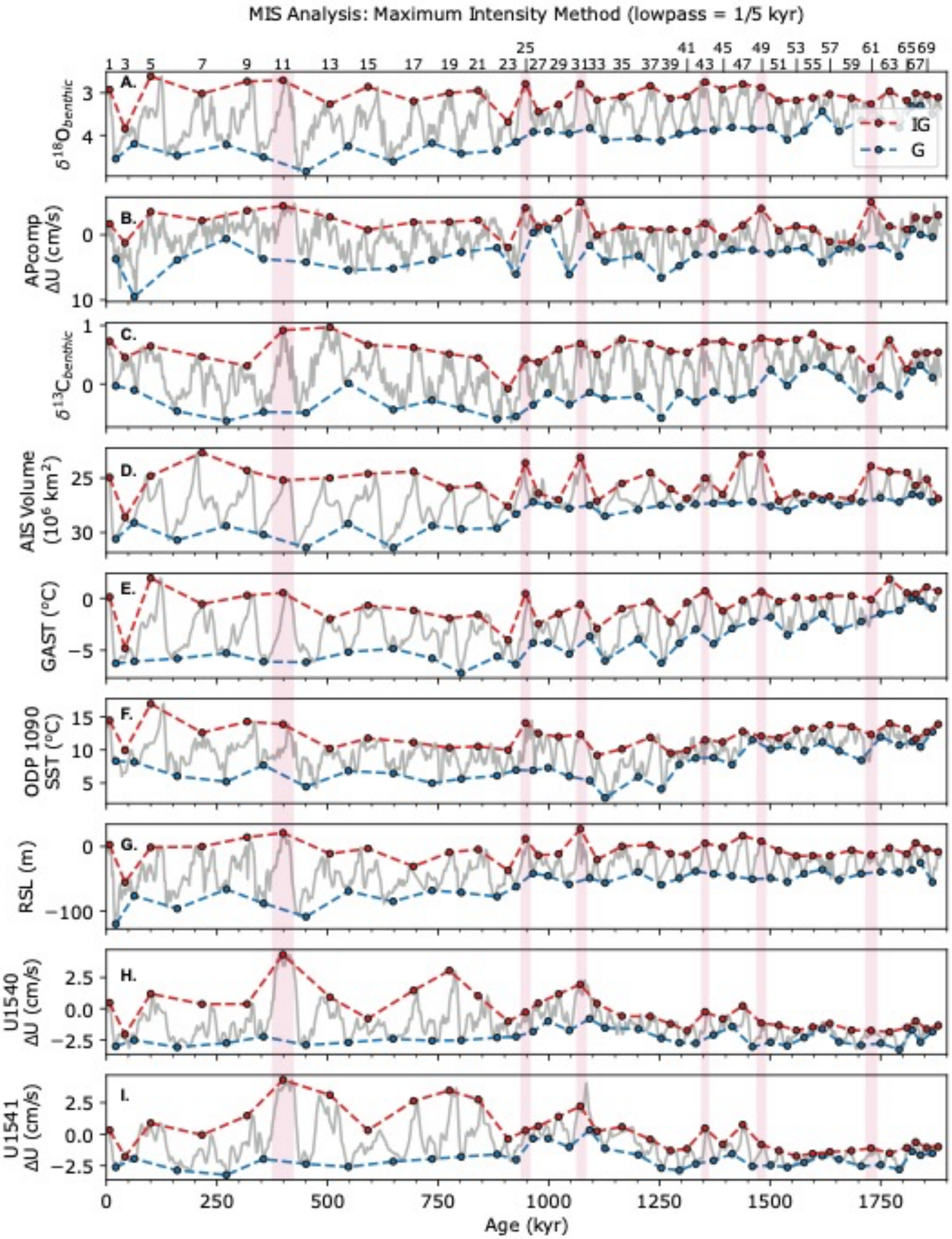

Fig. S7. Marine Isotope Stage analysis. Values determined for each MIS following the 'maximum intensity approach' for each record described in Supplementary Table 3 after applying a 1/5 kyr low pass filter. Glacial (even) stages are shown in blue and interglacial (red) stages are red.

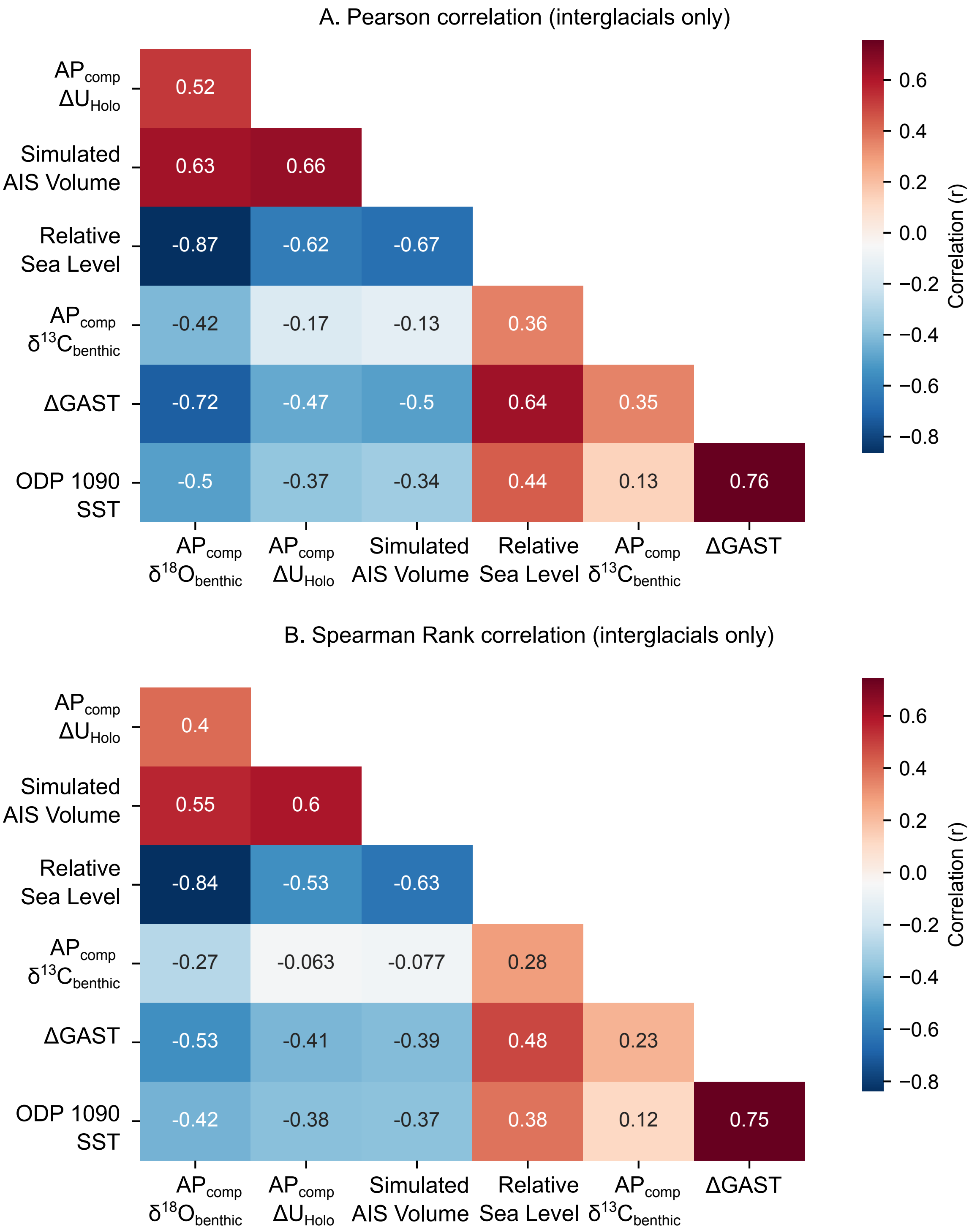

Fig. S8. Correlation statistics for MIS ensemble table (Supplementary Table 4) (for interglacial values). R is calculated using either Pearson's correlation statistic (a) or a Spearman Rank statistic (b).

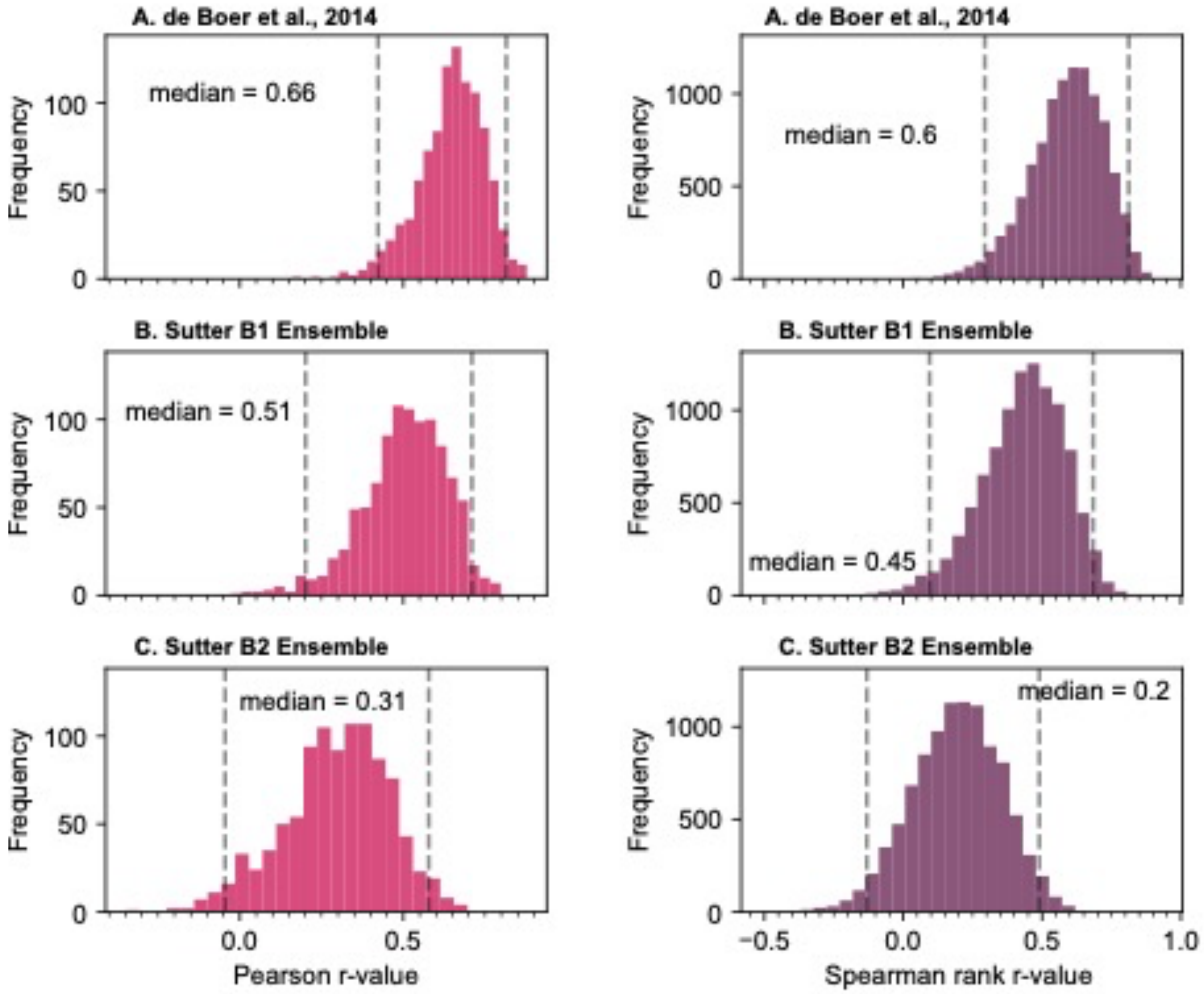

Fig. S9. Bootstrap correlation statistics for AP_{comp} SS versus various AIS volume models. Comparison of r-values for AP_{comp} SS against simulated AIS volume from the ref (75) model (top) and the B1 (middle) and B2 (lower) ensemble simulations of ref (76). Median r-value is given and the dashed lines represent 2.5% and 97.5% confidence intervals. Bootstrapping is performed by randomly sampling each dataset 10,000 times.

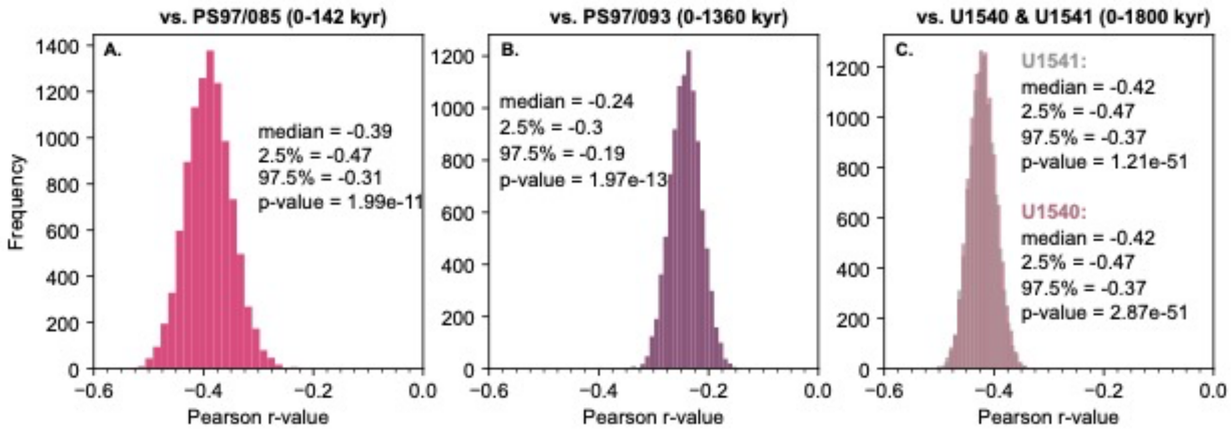

Fig. S10. Bootstrap correlation statistics for AP_{comp} SS versus Drake Passage and South Pacific SS records. (a) AP_{comp} versus PS097/085 ref (36) (interval 0 – 142 ka; both records linearly interpolated to 500 year spacing). (b) AP_{comp} versus PS097/093 (55) (0 – 1360 ka; both records linearly interpolated to 1.5 kyr spacing). (c) APcomp versus U1541 and U1540 (19) (0 – 1800 ka; both records linearly interpolated to 1.5 kyr spacing).

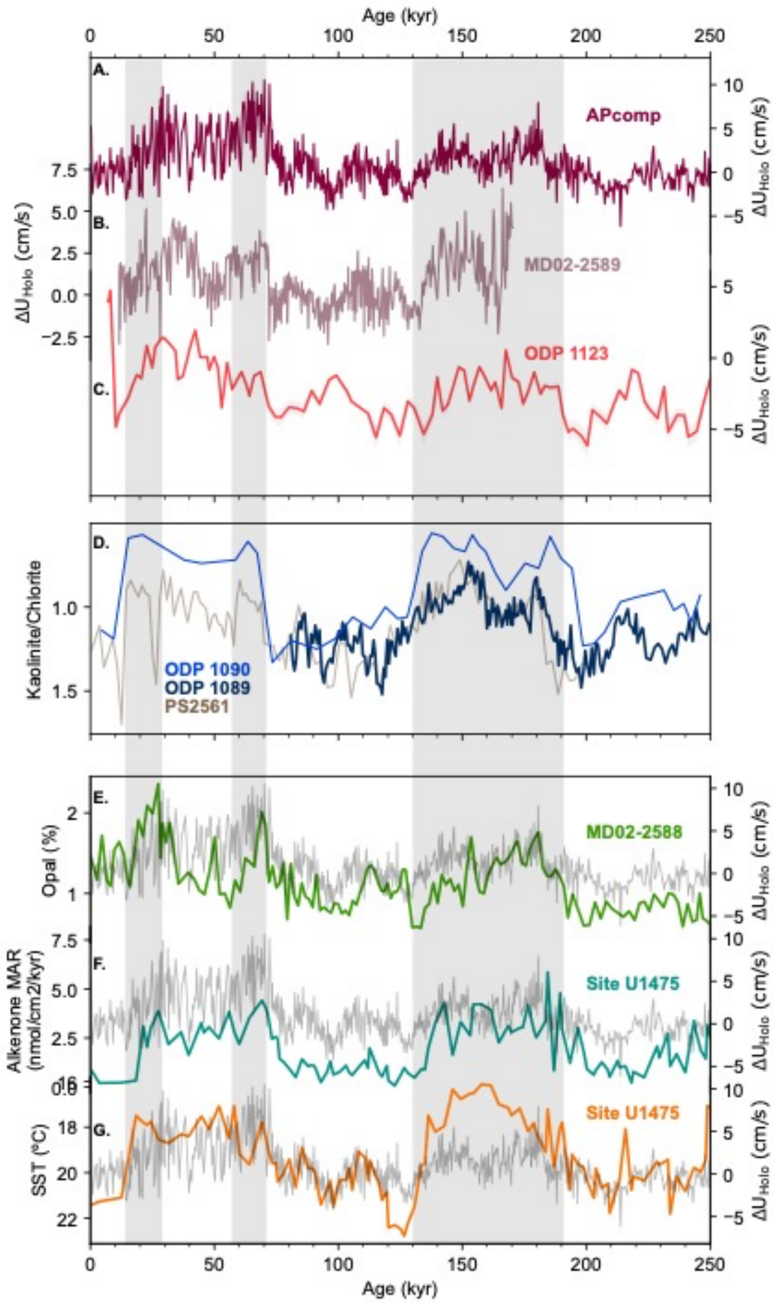

Fig. S11. Last glacial cycle ACC and SO front records. (a) APcomp ΔU_{Holo} (this study). (b) MD02-2589 ΔU_{Holo} (22). (c) ODP Site 1123 ΔU_{Holo} (33) (e) Kaolinite/Chlorite clay ratio from ODP Site 1090 (40), ODP Site 1089 (103), and PS2561 (39). (f) Opal (weight %) in MD02-2588 (111) (AP_{comp}). (g) Alkenone accumulation rate and (h) UK37 derived SST at Site U1475 (108). Grey lines in the background of f-h shows the APcomp ΔU_{Holo} data.

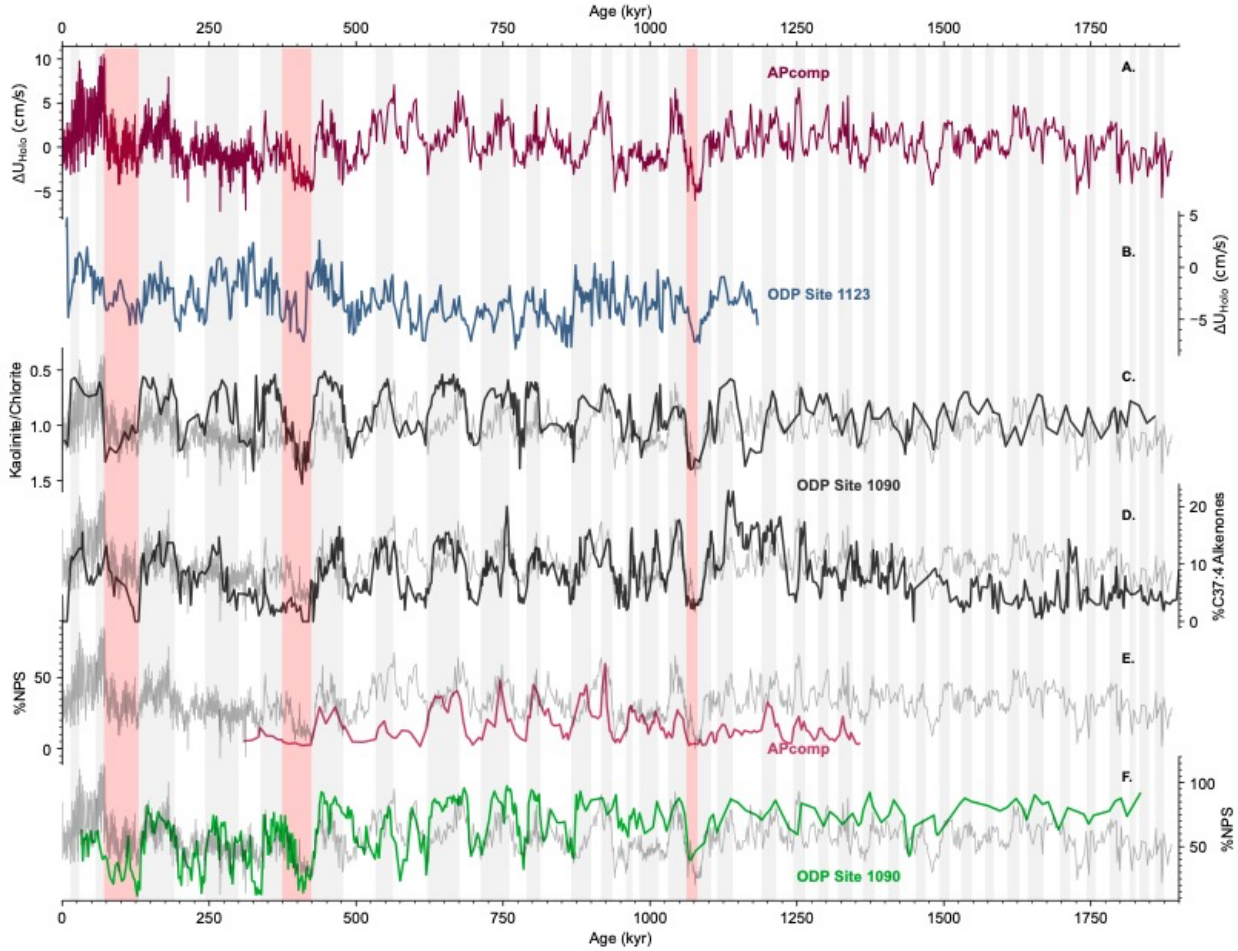

Fig. S12. Hydrographic and dynamic tracers of the ACC since 1.9 Ma. (a) APcomp ΔU_{Holo} (this study). (b) ODP Site 1123 $\Delta U_{Holo.}$ (33) (c) Kaolinite/Chlorite clay ratio from ODP Site 1090(40). (d) Portion of polar water alkenone biomarkers at ODP Site 1090. (e-f) % Abundance of *N. pachyderma* at (e) ODP Site 1090 (62) and (f) the AP_{comp} (97).

Record name	Description	Ref.	G/IG
AP_{comp} $\delta^{18}O_{benthic}$ (‰)	*C. wuellerstorfi* $\delta^{18}O$ record from the AP_{comp}	(20)	Min values for glacials
AP_{comp} ΔU_{Holo} (cm/s)	AP_{comp} Sortable Silt converted to % flow speed change from Holocene	*This study*	Max values for glacials
Simulated AIS volume (10^6 km^2)	Antarctic Ice Sheet volume simulated by the ANICE 3-D ice-sheet-shelf model	(75)	Max values for glacials
ΔGAST (°C)	Change in global average surface temperature (estimated through proxy record assimilation)	(73)	Min values for glacials
Relative Sea Level (RSL) (m)	Changes in RSL estimated with foraminiferal $\delta^{18}O$ and Mg/Ca records	(77)	Min values for glacials
ODP 1090 SST (°C)	UK37 SST from ODP Site 1090	(62)	Min values for glacials
IODP Site U1541 ΔU_{Holo} (cm/s)	U1541 Sortable Silt converted to % flow speed change from Holocene	(19)	Min values for glacials

Table S1. Metadata for original and published datasets used for MIS analysis in this study. 'G/IG' column notes whether maximum or minimum values are chosen for glacial intervals.

MIS	Time ($\delta^{18}O_{benthic}$ peak age)	AP_{comp} $\delta^{18}O_{benthic}$ (‰)	AP_{comp} ΔU_{Holo} (%)	Simulated AIS volume (10^6 km^2)	ODP Site 1090 SST (ºC)	DGAST (ºC)	RSL (m)
1	3.27	2.92	-6.20	24.93	14.47	0.13	2.8
2	19.27	4.55	13.75	30.60	8.31	-6.26	-120.4
3	55.27	3.85	4.61	28.60	9.92	-4.81	-55.7
4	69.27	4.20	35.15	29.10	8.16	-6.07	-76.7
5	125.27	2.61	-13.01	24.80	16.95	1.94	-1.4
6	139.27	4.48	14.25	30.70	6.02	-5.80	-96.5
7	215.27	3.02	-8.00	22.67	12.59	-0.56	-0.2
8	273.27	4.22	2.32	29.40	5.18	-5.27	-66.4
9	327.27	2.73	-13.68	24.30	14.27	0.28	14.3
10	345.27	4.52	13.72	30.20	7.68	-6.10	-88.3
11	407.27	2.70	-16.32	25.20	13.87	0.52	21.0
12	433.27	4.85	15.52	31.40	4.43	-6.15	-109.0
13	491.27	3.27	-10.00	25.00	10.19	-1.98	-11.2
14	537.27	4.26	20.04	29.20	6.82	-5.17	-69.3
15	613.27	2.86	-2.74	24.60	11.75	-0.69	-3.2
16	635.27	4.62	19.10	31.40	6.45	-4.84	-85.4
17	697.27	3.20	-7.08	24.40	11.12	-1.17	-31.0
18	721.27	4.18	14.33	29.40	4.97	-5.77	-68.3
19	781.27	3.00	-7.31	25.90	10.31	-1.94	-9.1
20	795.27	4.43	9.84	29.70	5.61	-7.20	-71.5
21	857.27	2.95	-8.21	25.70	10.53	-1.59	-4.6
22	881.27	4.36	7.38	29.60	6.10	-5.59	-77.9
23	911.27	3.69	7.26	27.60	9.97	-4.02	-37.4
24	921.27	4.16	22.29	28.30	6.95	-6.35	-62.4
25	955.27	2.79	-15.24	23.60	14.09	0.45	12.2
26	969.27	3.92	-1.19	27.20	6.90	-4.28	-41.7
27	977.27	3.45	-4.54	26.40	12.50	-2.46	-13.5
28	1007.27	3.91	-3.30	27.50	7.29	-4.27	-45.6
29	1025.27	3.28	-9.09	27.00	11.97	-1.47	-11.8
30	1037.27	3.96	22.57	27.80	6.00	-5.36	-58.7
31	1077.27	2.79	-18.56	23.10	12.34	-0.57	27.1
32	1099.27	3.83	6.01	27.50	5.39	-3.68	-49.1
33	1109.27	3.17	0.20	27.10	9.18	-2.90	-20.6
34	1123.27	4.11	15.00	28.50	2.73	-6.01	-56.5
35	1157.27	3.09	-4.58	25.50	10.12	-1.01	0.3
36	1201.27	4.07	11.86	27.90	5.92	-3.92	-39.6
37	1237.27	2.83	-2.88	24.50	11.89	-0.38	2.2
38	1251.27	4.13	24.35	27.50	4.06	-6.22	-59.6

39	1271.27	3.13	-3.04	26.00	9.50	-2.30	-11.0
40	1291.27	3.97	17.62	27.70	7.83	-4.32	-49.3
41	1315.27	3.09	-2.08	26.90	9.89	-0.38	-12.9
42	1331.27	3.90	11.15	27.40	8.75	-2.99	-38.4
43	1347.27	2.75	-6.46	25.00	11.49	0.70	5.0
44	1379.27	3.88	11.30	27.30	8.85	-4.38	-42.8
45	1389.27	2.92	1.36	26.50	11.17	-1.23	-1.3
46	1415.27	3.81	8.45	27.30	7.77	-2.91	-46.1
47	1447.27	2.79	-5.43	22.90	12.79	-0.20	16.8
48	1457.27	3.85	8.73	27.20	11.51	-2.23	-50.7
49	1473.27	2.88	-14.91	22.77	12.07	0.64	8.0
50	1503.27	3.82	10.62	27.60	10.08	-1.80	-49.2
51	1527.27	3.19	-2.25	27.10	11.80	-0.31	-6.4
52	1541.27	4.10	8.49	28.00	10.57	-3.53	-55.2
53	1561.27	3.18	-4.88	26.40	13.05	0.10	-15.1
54	1579.27	3.89	7.27	27.30	9.84	-2.74	-42.1
55	1603.27	3.12	-3.46	26.60	13.28	-0.01	-14.4
56	1613.27	3.43	15.81	27.00	11.17	-1.50	-35.7
57	1635.27	3.03	3.89	26.70	13.74	0.21	-14.6
58	1663.27	3.91	8.03	27.50	9.83	-3.06	-52.1
59	1679.27	3.12	4.56	26.90	13.51	0.25	-5.7
60	1705.27	3.65	7.51	27.20	8.43	-2.25	-42.3
61	1717.27	3.27	-18.54	23.90	12.31	-0.12	-12.9
62	1745.27	3.62	6.11	26.80	11.97	-1.46	-39.1
63	1763.27	2.96	-4.78	24.40	13.98	1.87	-2.3
64	1795.27	3.83	12.17	27.20	10.70	-1.18	-40.6
65	1813.27	3.19	-2.95	24.50	13.20	0.51	-11.8
66	1823.27	3.32	-3.08	26.50	11.23	0.01	-36.2
67	1827.27	3.01	-9.87	25.70	11.68	0.42	5.5
68	1835.27	3.30	-0.08	26.60	10.44	-0.25	-25.4
69	1849.27	3.05	-8.62	25.10	12.71	1.08	-3.7
70	1867.27	3.50	1.38	27.20	12.75	-0.94	-55.4
71	1875.27	3.11	-11.05	26.90	13.88	0.69	-8.4

Table S2. Marine Isotope Stage ensemble table estimated using the 'Maximum Intensity Approach'. 'Time' is given as the time (on the LR04 timescale) of the AP_{comp} $\delta^{18}O_{benthic}$ peak (or trough) associated with the given MIS. Metadata for each record is given in Supplementary Table 3). Only selected records are shown here, the full ensemble is available in the supplementary code (https://github.com/AidanStarr/Starr_etal_ACC)
